# A randomized, placebo‐controlled, dose‐escalation phase I/II multicenter trial of low‐dose cidofovir for BK polyomavirus nephropathy

**DOI:** 10.1111/tid.14367

**Published:** 2024-09-03

**Authors:** Hannah Imlay, John W. Gnann, James Rooney, V. Ram Peddi, Alexander C. Wiseman, Michelle A. Josephson, Clifton Kew, Jo‐Anne H. Young, Deborah B. Adey, Milagros Samaniego‐Picota, Richard J. Whitley, Ajit P. Limaye

**Affiliations:** ^1^ Department of Internal Medicine University of Utah Salt Lake City Utah USA; ^2^ Department of Medicine Medical University of South Carolina University Medical Center Charleston South Carolina USA; ^3^ Gilead Sciences Foster City California USA; ^4^ Department of Transplantation California Pacific Medical Center San Francisco California USA; ^5^ Department of Medicine University of Colorado at Denver Health Sciences Center Denver Colorado USA; ^6^ Department of Medicine University of Chicago Chicago Illinois USA; ^7^ Department of Medicine University of Alabama at Birmingham Birmingham Alabama USA; ^8^ Department of Medicine University of Minnesota Minneapolis Minnesota USA; ^9^ Department of Medicine University of California at San Francisco San Francisco California USA; ^10^ Department of Medicine University of Wisconsin Medical School Madison Wisconsin USA; ^11^ Department of Pediatrics University of Alabama Birmingham Alabama USA; ^12^ Department of Internal Medicine University of Washington Seattle Washington USA

**Keywords:** BK virus, kidney transplant, cidofovir, randomized controlled trial

## Abstract

**Background:**

BK polyomavirus‐associated nephropathy (BKPyVAN) is an important cause of allograft dysfunction and failure in kidney transplant recipients (KTRs) and there are no proven effective treatments. Case reports and in vitro data support the potential activity of cidofovir against BK polyomavirus (BKPyV).

**Methods:**

We report the results of a phase I/II, double‐blind, placebo‐controlled randomized dose‐escalation trial of cidofovir in KTRs with biopsy‐confirmed BKPyVAN and estimated glomerular filtration rate ≥30 mL/min. Intravenous cidofovir (0.25 mg/kg/dose or 0.5 mg/kg/dose) or placebo was administered on days 0, 7, 21, and 35, with final follow‐up through day 49.

**Results:**

The trial was prematurely discontinued due to slow accrual after 22 KTRs had completed the study. Cidofovir was safe and tolerated at the doses and duration studied. The proportion of subjects with any adverse event (AE) was similar between groups (9/14 [64%] in the combined cidofovir dose groups and 6/8 [75%] in the placebo group); 84% of AEs were mild. BKPyV DNAemia reduction by day 49 was similar between groups (>1 log_10_ reduction in (2/9 [22.2%] of 0.25 mg/kg group, 1/5 [20%] of 0.5 mg/kg group, and 2/8 [25%] of placebo group).

**Conclusions:**

These preliminary results indicate that low‐dose cidofovir was safe and tolerated but had no significant BKPyV‐specific antiviral effect in KTRs with BKPyVAN.

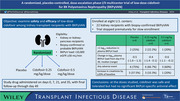

## Graphical abstract

In a randomized, placebo‐controlled, dose‐escalation phase I/II multicenter trial of low‐dose cidofovir for BK polyomavirus nephropathy, cidofovir was found to be safe and tolerated but demonstrated no significant BK polyomavirus‐specific antiviral effect.

**Abbreviations**
AE: adverse event
ANC: absolute neutrophil count
BKPyV: BK polyomavirus
BKPyVAN: BK polyomavirus‐associated nephropathy
CASG: collaborative antiviral study group
DSMB: Data Safety and Monitoring Board
eGFR: estimated glomerular filtration rate
KTR: kidney transplant recipient
MDRD: modification of diet in renal disease
MTD: maximum tolerated dose
NIAID: National Institute of Allergy and Infectious Diseases
NIH: National Institutes of Health
PD: pharmacodynamic
PK: pharmacokinetic

## 1 INTRODUCTION

BK polyomavirus (BKPyV)‐associated nephropathy (BKPyVAN) is a major cause of renal injury and graft loss among kidney transplant recipients (KTRs). BKPyVAN is characterized by pathologic changes in renal tissue and is typically accompanied by high‐level BKPyV DNAemia. A plasma BKPyV DNAemia threshold of >10,000 copies/mL has been proposed as “presumptive BKPyVAN” based on its correlation in some assays with histologic evidence of BKPyVAN.^1^, ^2^, ^3^ Progression to BKPyVAN continues to occur despite reductions in immunosuppression guided by surveillance testing.^1^, ^4^ Further, immunosuppression reduction can be complicated by the development of acute cellular rejection or donor‐specific antibodies.^5^, ^6^, ^7^ Thus, specific antiviral therapies are needed.

Antiviral therapies for the prevention or treatment of BKPyVAN have been evaluated but have not been effective in randomized controlled trials or approved for this indication, including fluoroquinolones and FK778.^8^, ^9^, ^10^ Cidofovir is a monophosphate nucleotide analog that has demonstrated in vitro activity against BKPyV and, in some observational studies, has been associated with a reduction in BKPyV DNAemia.^4^, ^11^, ^12^, ^13^ Cidofovir is approved for the treatment of HIV‐associated CMV retinitis and has been used for CMV DNAemia and disease among other immunocompromised patients at doses of 5 mg/kg every 1–2 weeks, given with probenecid.^14^, ^15^ Cidofovir is also associated with nephrotoxicity and other adverse effects that limit its use.^16^, ^17^, ^18^, ^19^, ^20^ Cidofovir has not previously been evaluated in a controlled, randomized trial for the treatment of BKPyVAN. This study, sponsored by the National Institute of Allergy and Infectious Diseases (NIAID), and the National Institutes of Health (NIH), was a randomized multicenter trial of cidofovir among adults with BKPyVAN. The study was discontinued prior to full accrual because of slower‐than‐anticipated enrollment.

## 2 METHODS

### 2.1 Study design

This was a randomized, multicenter, double‐blind, placebo‐controlled, sequential dose‐escalation trial to evaluate the safety, tolerability, and preliminary efficacy of IV cidofovir in KTRs with newly diagnosed BKPyVAN. The study was planned as a dose escalation study with three dose cohorts (0.25, 0.5, and 1.0 mg/kg) with a proposed enrollment of 12 patients per cohort, randomized 2:1 in parallel groups to cidofovir versus placebo (0.9% normal saline). Once a maximum tolerated dose (MTD) was established, 12 additional patients were anticipated to be enrolled at the MTD for efficacy analyses.

The NIH, institutional review boards at all sites, and an independent Data Safety and Monitoring Board (DSMB) approved the study, which was registered on clinicaltrials.gov (NCT00138424). Written informed consent was obtained from all patients or their legally authorized representatives. Due to slower‐than‐anticipated subject accrual, the DSMB determined that the study could not fully accrue within a reasonable time span. Thus, enrollment was terminated mid‐way through Cohort II, after 22 subjects completed the study; no patients were enrolled into Cohort III (1.0 mg/kg); additionally, funding and trial oversight of the collaborative antiviral study group (CASG) was discontinued by NIAID.

### 2.2 Patients

Eligible patients were kidney or kidney‐pancreas transplant recipients age ≥18 years with newly diagnosed BKPyVAN, defined as either “biopsy‐confirmed” (renal biopsy demonstrating BK virus by immunohistochemistry, electron microscopy, or in situ hybridization) or “probable” (BKPyV load in plasma >10,000 copies/mL) within the prior 60 days. We use the term “probable BKPyVAN” to be consistent with consensus definitions for clinical trials^2^ however “presumptive BKPyVAN” has also been used to refer to plasma BKPyV DNAemia >10,000 copies/mL in major clinical practice guidelines.^1^, ^21^, ^22^ Patients with biopsy‐confirmed BKPyVAN were also required to have a BKPyV‐DNAemia in plasma >10,000 copies/mL within 21 days prior to enrollment (assay performed at local or central laboratories). Other key inclusion criteria included estimated glomerular filtration rate (eGFR) >30 mL/min (calculated by the original extended modification of diet in renal disease [MDRD] equation)^23^ and absolute neutrophil count (ANC) >1000/microL. Key exclusions included: previous treatment with cidofovir within the preceding 2 weeks, recent or planned receipt of other known nephrotoxic medications, contraindication to renal biopsy (for the purpose of subsequent evaluation), or coexisting ocular hypotony or uveitis (see Protocol in Supporting Information for full study procedures).

After subjects were enrolled, BKPyV DNA in plasma and urine was assessed at the CASG Central Laboratory (University of Alabama at Birmingham). Following precedent for BKPyV and other transplant viruses, we refer to the detection of viral load from plasma as “BKPyV‐DNAemia”.^1^, ^24^ Cidofovir dosing was adjusted for GFR per the Food and Drug Administration label. eGFR was calculated using the extended MDRD equation^23^ (Protocol in Supporting Information).

### 2.3 Randomization and study procedures

The study was planned to include 3 dose cohorts (0.25, 0.5, and 1.0 mg/kg) with each subject receiving a total of four doses on days 0, 7, 21, and 35. The study drug was initiated within 60 days of the diagnosis of BKPyVAN. Each cohort was planned to include 12 subjects randomized 2:1 using web‐based randomization to receive either cidofovir or placebo (0.9% normal saline). The sample size was chosen based on feasibility considerations and a 15.3% estimated standard error for adverse event (AE) rate for each treatment group (*n* = 8) and a 21.7% estimated standard error for AE rate in each control group (*n* = 4), assuming an AE rate of ≤25% (Protocol in Supporting Information).

Urine and plasma BKPyV samples for quantitative polymerase chain reaction were obtained during the 3‐week screening period, within 48 h of dosing on day 0, and then at follow‐up visits on days 7, 21, 35, and 49; samples were stored at −80°C at enrolling sites and batch shipped to the Central Laboratory (assessed as previously published at CASG laboratory^25^). BKPyV‐DNA loads were reported in copies/mL (conversion: 1 IU = 1.72 copies/mL). Physical examination, review for AEs, and laboratory assessments were performed at all visits. Pharmacokinetic (PK) and pharmacodynamic (PD) assessments were additionally performed. The results of PK/PD analyses are included in subsections 15 and 16 of the final study report on clinicaltrials.gov.

Immunosuppression was reduced at the time of BKPyVAN diagnosis at the discretion of the patient's clinical team; adjustments were individualized but generally followed guidance provided in the study protocol, which suggested reduction of mycophenolate mofetil dose by increments of 250 mg twice daily every 2 weeks to a baseline dose of 500 mg twice daily to 0 mg daily ± calcineurin inhibitor dose reduction (tacrolimus dose adjusted for goal trough level of 5–7 ng/mL).

### 2.4 Outcomes

The primary study outcomes were safety and tolerability of cidofovir at the studied doses and virologic efficacy assessments measured at end‐of‐treatment on day 49.

#### 2.4.1 Safety and tolerability

The prespecified outcomes were deterioration in renal function, decline in ANC to <500 cells/microL, and ocular complications (hypotony, uveitis). Any subject who developed a >50% reduction in eGFR from baseline underwent renal biopsy as part of routine clinical care and the study drug was discontinued. Additional endpoints included ≥25% decrease in eGFR or ≥20% increase in creatinine.

#### 2.4.2 Efficacy

The primary efficacy endpoint was a ≥1‐log decrease in BKPyV‐DNAemia measured at the central laboratory between baseline and day 49. Additional prespecified analyses included the percentage of study participants who achieved a negative BKPyV urine or plasma DNA result between baseline and day 49 and the percentage of study participants who had any reduction in urine/plasma BKPyV DNA level between baseline and day 49.

### 2.5 Sample size and statistical analysis

Each cohort was planned to include 12 subjects. Virologic endpoints and AEs were reported descriptively. SAS 9.0 and Rstudio 1.4.1106 were used for statistical assessments and the generation of figures.

## 3 RESULTS

### 3.1 Study population

**FIGURE 1:**
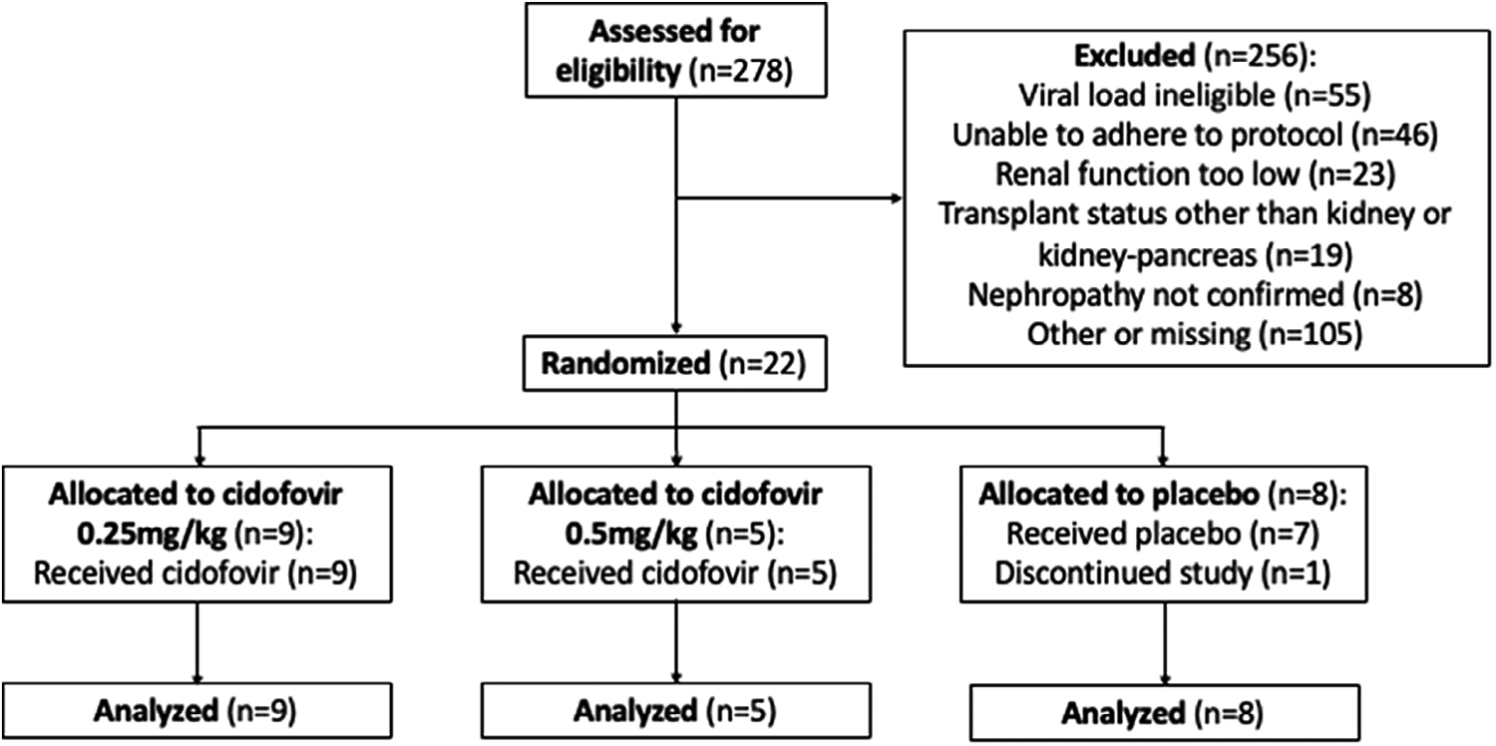
Flow diagram of patient enrollment.

A total of 278 patients with new onset BKPyVAN were screened and 22 (7.9%) were enrolled (Figure 1; Appendix S1) between 5/22/2006 and 5/11/2010. The most common reasons for lack of enrollment included a lack of qualifying DNA load measurement, inability to adhere to the trial protocol or eGFR of <30 mL/min. All enrolled subjects were kidney recipients alone and all subjects had biopsy‐confirmed BKPyVAN diagnoses at a median of 25.5 days before enrollment (interquartile range of 14.75–34.75). Nine patients received cidofovir (0.25 mg/kg), five received cidofovir (0.5 mg/kg), and eight received a placebo and completed the study before the trial was terminated by NIAID. The demographics of enrolled patients are listed in Table 1.

**TABLE 1:** Demographics and clinical characteristics of cohort.

	Cidofovir 0.25 mg/kg *n* = 9	Cidofovir 0.5 mg/kg *n* = 5	Cidofovir (combined cohorts) *n* = 14	Placebo (combined cohorts) *n* = 8
White race, non‐Hispanic (%)	5 (55.5)	2 (40)	7 (50)	5 (62.5)
Self‐identified Male sex (%)	6 (66.7)	5 (100)	11 (79)	7 (87.5)
Age at enrollment, median years (IQR)	57 (53‐65)	60 (53−66)	58.5 (53−65)	46.5 (38.8−59.5)
Time since transplant, median months (IQR)	22 (7.8−29.2)	8.5 (7.9−48.9)	15.5 (7.8−29.5)	21.3 (8.9−35.0)
Deceased donor transplant (%)	7 (77.8)	3 (60)	10 (71)	5 (62.5)
Creatinine at enrollment, median mg/dL (IQR)	1.5 (1.4−2.2)	1.5 (1.1−1.7)	1.5 (1.2−1.8)	1.4 (1.2−1.7)
eGFR at enrollment^a^, median (IQR)	44 (37−52)	48 (40−79)	46 (37−57)	57 (43−68)
Baseline absolute lymphocyte count <500 (%)^b^	1/5 (20)	0/5 (0)	1/10 (10)	2/5 (40)
Baseline immunosuppression^c^:				
Tacrolimus/mycophenolate/prednisone	3/7 (43)	4/5 (80)	7/12 (6)	6/8 (75)
Tacrolimus/prednisone	2/7 (29)	0	2/12 (17)	0
Tacrolimus/mycophenolate	1/7 (14)	0	1/12 (8)	2/8 (25)
Other^d^	1/7 (14)	1/5 (20)	2/12 (17)	0
Tacrolimus trough, median (IQR)^e^	5.7 (5.6−7.8)	6 (3.9−7.6)	5.7 (3.9−7.7)	5.8 (5.1−7.6)

^a^ Calculated using an extended MDRD equation (see study protocol).

^b^ Data for seven patients were missing.

^c^ Data for two patients were missing.

^d^ Other consists of tacrolimus/sirolimus/prednisone (*n* = 1), and sirolimus/mycophenolate/prednisone (*n* = 1).

^e^ Data available for 5/8 patients in the placebo group, 6/9 patients in the 0.25 mg/kg cidofovir group, and 3/5 patients in the 0.5 mg/kg cidofovir group.

### 3.2 Safety and tolerability

No specific AE occurred at a high rate. Fifteen of 22 subjects (68%) had at least one AE (6/8 [75%] of patients in the placebo group and 9/14 [64%] in the cidofovir groups); the majority (83.9%) were mild (Table 3). Thirteen percent of the AEs were attributed to the study drug (4/8 [50%] in the placebo group vs. 5/14 [36%] in the intervention group). Two serious AEs were reported, both in the placebo group (exacerbation of cellulitis and acute cellular rejection); one was judged by the investigator to be related to treatment. One participant in the placebo arm discontinued the study early due to an elevation of creatinine and volume overload that was deemed by the investigator to be potentially related to treatment. No deaths or graft losses occurred among study subjects by day 49 (end of study follow‐up).

There was no significant reduction in white blood cell count or ANC between Day 0 and Day 49 in either dosing cohort. Routine chemistries and liver function were not significantly different between the two groups. No differences in chemistry and urinalysis, including serum creatinine and eGFR, were noted between Day 0 and Day 49, or between the two treatment groups (Table 3).

### 3.3 Efficacy

**FIGURE 2:**
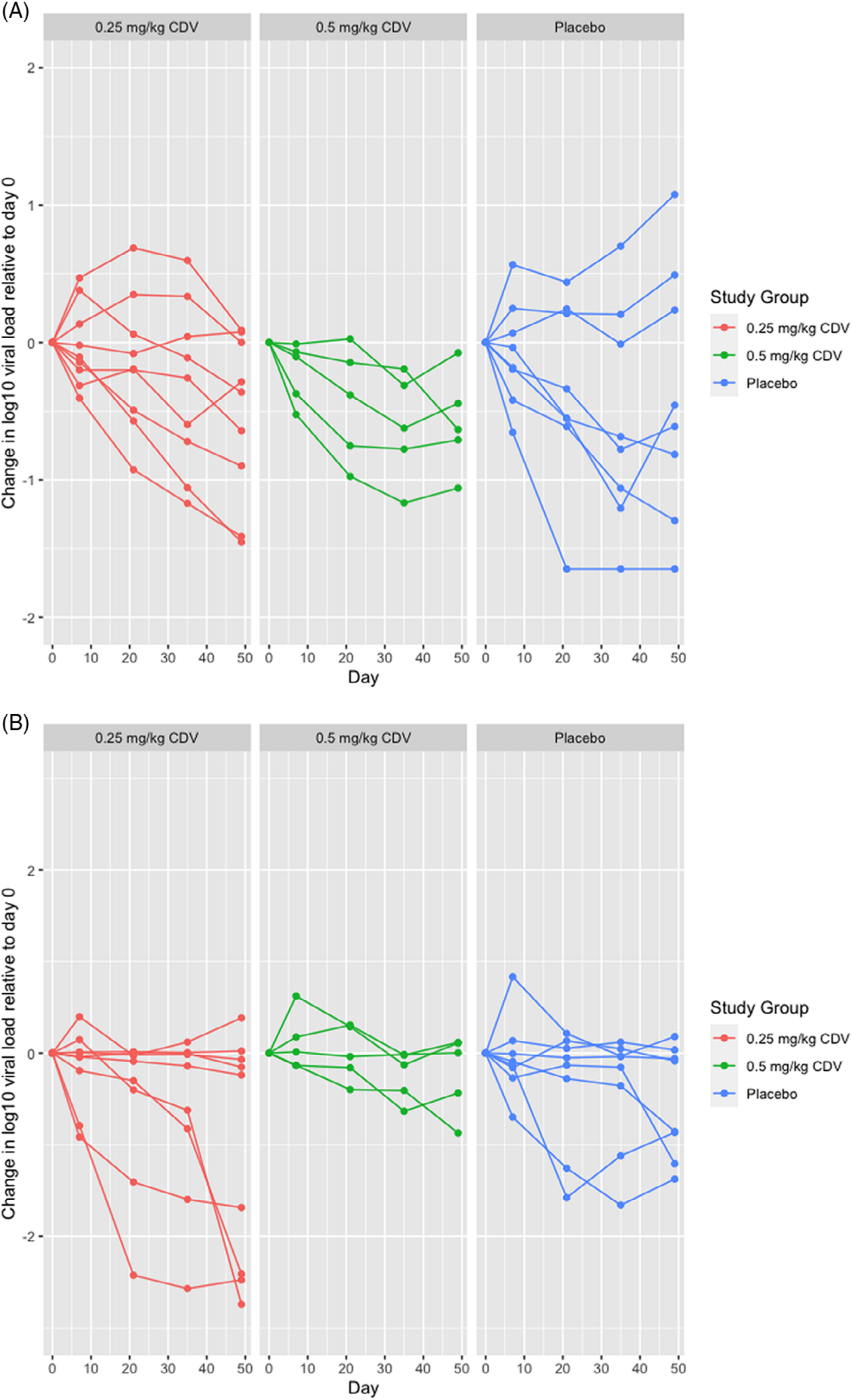
(A, B) Individual patient log_10_ plasma DNA load over time normalized to baseline BK polyomavirus (BKPyV) DNA load (A); individual urine log_10_ DNA loads over time normalized to baseline urine BKPyV DNA load (B).

Median plasma and urine BKPyV DNA loads at day 0 were: 5.4 log_10_ copies/mL (range 4.0–7.5) and 8.9 log_10_ copies/mL (range 4.5–9.6), respectively (Table 2). Changes in BKPyV DNA levels in the treatment and placebo groups showed similar trends (Figure 2A,B and Figures S1–S4). The only subject who achieved undetectable BKPyV‐DNAemia (i.e., <200 copies/mL, the limit of detection of the assay) was in the placebo arm (baseline plasma BKPyV‐DNAemia 3.9 log_10_ copies/mL). Only 5/22 (22.7%) of participants had a reduction in BKPyV‐DNAemia by ≥1 log_10_ by day 49: 2/8 in the placebo group, 2/9 in the 0.25 mg/kg group, and 1/5 in the 0.5 mg/kg group.

**TABLE 2:** Virologic data and endpoints.

	Cidofovir 0.25 mg/kg n D;= 9 (%)	Cidofovir 0.5 mg/kg n = 5 (%)	Placebo n = 8 (%)
**Plasma BKPyV DNAemia**			
Log_10_ VL at day 0, median (range)	5.9 (4.0–7.5)	5.3 (4.2–5.8)	5.3 (4.0–6.5)
Number with undetectable BKPyV load at day 49	0	0	1 (12.5)
Change in log_10_ VL from day 0 to day 49^a^, median (range)	−0.4 (−1.4–0.1)	−0.6 (−1.1–−0.1)	−0.5 (−1.6−1.1)
Number whose VL decreased by ≥1 log between day 0 to day 49	2 (22.2)	1 (20)	2 (25)
**Urine DNAuria**			
Log of VL at day 0, median (range)	9.1 (4.5–9.6)	8.6 (5.9–9.1)	8.8 (5.8–9.3)
Number with undetectable BKPyV load at day 49	0	0	0
Change in log VL from day 0 to day 49^a^, median (range)	−0.2 (−2.7−0.4)	0.0 (−0.9−0.1)	−0.5 (−1.4−0.2)
Number whose VL decreased by ≥1 log from day 0 to day 49	4 (44.4)	0	2 (25)

^a^ A negative result signifies a decrease in viral load on day 49 relative to day 0.

**TABLE 3:** Safety endpoints.

	Cidofovir 0.25 mg/kg *n* = 9 (%)	Cidofovir 0.5 mg/kg *n* = 5 (%)	Placebo *n* = 8 (%)
Patients with ≥1 adverse event	6 (66.7)	3 (60)	6 (75)
Patients with ≥1 serious adverse event^a^	0	0	2 (25)
WBC count <3500 cells/uL compared to baseline	2 (22)	1 (20)	1 (13)
ANC<1000 cells/uL^a^	1 (11)	0	0
Grade 2‐4 ocular effects during the study period	0	0	0
Development of acute cellular rejection	0	0	1 (13)
Graft loss	0	0	0
**Creatinine change**			
Increase in creatinine, median (IQR)	0 (−0.1, 0.2)	0 (−0.1, 0.0)	0.2 (0, 0.3)
Increase in creatinine, mean (std dev)	0.17 (0.5)	0 (0.21)	0.2 (0.2)
Increase in creatinine by ≥20% of baseline	3 (33.3)	1 (20)	3 (37.5)
**eGFR change**			
Change in eGFR, median (IQR)	−2.3 (−7.5, 4.8)	−1.3 (−1.8, 1)	−3 (−13.9, 2.5)
Change in eGFR, mean (std dev)	−3.5 (9.5)	−1.3 (6.0)	−4.3 (9.5)
Decrease in eGFR by ≥50% of baseline	1 (11)	0	0
Decrease in eGFR by ≥25% of baseline	1 (11)	0	1 (12.5)

^a^ No patients had ANC < 500.

## 4 DISCUSSION

In this randomized, placebo‐controlled trial among KTRs with biopsy‐confirmed BKPyVAN, within the limitations of the small sample size, low‐dose cidofovir was safe and well‐tolerated but had no significant effect on BKPyV DNA levels in either urine or blood. These data do not support cidofovir as an effective treatment for BKPyVAN in KTR at the low doses studied.

Cidofovir has been associated with multiple potential adverse effects, including nephrotoxicity, ocular hypotonia, uveitis, and leukopenia.^16^, ^17^, ^18^, ^19^, ^20^ Despite frequent clinical and laboratory assessments during the intervention period, this study identified a low frequency of AEs with cidofovir at the dose regimens used. The optimal dose of cidofovir was not determined in the study, but typical doses used in prior studies for the treatment of BKPyV‐DNAemia or BKPyVAN in KTRs have ranged from 0.25 to 1 mg/kg/dose every 1–3 weeks without probenecid.^1^ Concurrent probenecid was not used in this study based on the lack of an effect on cidofovir plasma concentrations at doses <1 mg/kg.^26^ Although the full dose‐ranging protocol was not completed for this study, no major safety concerns were evident with these regimens in the small number of patients during the relatively short follow‐up period.

Cidofovir has been used as antiviral therapy in BKPyVAN but there are no supportive data from placebo‐controlled trials. At the low doses studied, there was no significant effect of cidofovir on BKPyV DNA levels in blood or urine in either of the cidofovir dose groups compared to placebo. Clearance of BKPyV‐DNAemia occurred in only 1 placebo participant during the 7‐week study period, which is consistent with other studies demonstrating clearance of BKPyV DNAemia after a mean of 6–12 months^27^, ^28^ among KTRs with biopsy‐confirmed BKPyVAN. The expected decline in BKPyV load with a clinically effective antiviral agent is unknown, and a virologic endpoint is not currently considered appropriate as a primary outcome in registrational (phase 3) interventional clinical trials by regulatory authorities.^2^ However, efficacious antivirals would likely affect plasma BKPyV load; BKPyV‐DNAemia results from BKPyV replication within the allograft,^3^, ^28^ and the level of BKPyV‐DNAemia is quantitatively associated with biopsy‐confirmed BKPyVAN.^1^, ^3^, ^27^, ^29^, ^30^, ^31^, ^32^, ^33^, ^34^ Funk et al modeled viral replication and concluded that a 50% reduction of BKPyV in the allograft would be associated with a decline in plasma BKPyV‐DNAemia of 1–2 log_10_ units, although would not result in clearance.^35^ An effective antiviral agent should at minimum be expected to result in a >1 log decline in plasma BKPyV load over 4–8 weeks.^35^, ^36^, ^37^, ^38^, ^39^, ^40^, ^41^, ^42^, ^43^ By multiple objective measures of BKPyV‐DNAemia reduction (proportion with >1 log reduction, proportion with resolution of BKPyV‐DNAemia), there was no antiviral effect of cidofovir at the doses and regimens used beyond what was achieved by reducing immunosuppression alone (i.e., placebo group). However, routine follow‐up biopsies to assess the extent of BKPyV in the allograft were not performed in this study and it is possible that higher doses of cidofovir might have higher efficacy.

An important strength of this study is that it is the only randomized, placebo‐controlled trial of cidofovir for the treatment of BKPyVAN in KTR. The use of frequent and standardized BKPyV‐DNA measurements performed in a central laboratory by blinded personnel allowed for detailed and unbiased characterization of changes in BKPyV DNA levels in urine and blood during the treatment period. We acknowledge important limitations. The study was small and there were differences in baseline characteristics between groups, which limits robust statistical comparative assessment of cidofovir dosing or cidofovir use versus placebo. We cannot determine whether higher doses of IV cidofovir would have been more effective or toxic. The follow‐up interval was short, which may have lowered the observed rate of nephrotoxicity and other AEs. Due to the potential for nephrotoxicity associated with cidofovir, this study enrolled only patients who had an eGFR of ≥30 mL/min, and thereby excluded many patients with new‐onset BKPyVAN who may present with more severe renal dysfunction^3^; other major BKPyV‐specific reasons for exclusions include requirement of specific BKPyV load and requirement for renal allograft biopsy. We used doses that had previously been tolerated and associated with a BKPyV antiviral effect, and we are unable to say whether higher doses may have had an antiviral effect or increased the risk of toxicity. Biopsies were unable to be classified using more recent Banff grading. Reductions in immunosuppression were per clinical practice rather than protocol mandated but are unlikely to have impacted BKPyV DNA levels over the short time frame of follow‐up.

Effective and safe antiviral therapy remains an important unmet need for BKPyVAN in KTRs. In this randomized, placebo‐controlled trial of KTRs with biopsy‐confirmed BKPyVAN who underwent standard immunosuppression reduction, the low doses of cidofovir did not lead to a significant decline in BKPyV DNA levels in urine or blood over 7 weeks. No major safety concerns related to low‐dose cidofovir administration were evident during the short duration of follow‐up. Based on these preliminary results, cidofovir at the low doses studied cannot be recommended as an effective therapy for BKPyVAN in KTR.

## CONFLICT OF INTEREST STATEMENT

Disclosures include: research grant from MEMO therapeutics [Clifton Kew]; consulting fees from Sanofi, Veloxis, Transplant Genomics [Alexander C. Wiseman]; speaker fees from Sanofi, Veloxis, Takeda [Alexander C. Wiseman], employee and stockholder at Gilead Sciences [James Rooney]. The remainder of the authors declare no conflict of interest.

## Supporting information

Supporting information

Supporting information

Visual Abstract Supporting information

## Data Availability

The data that support the findings of this study are available from the corresponding author upon reasonable request.
