# Supplementary material for: A randomized, placebo‐controlled, dose‐escalation phase I/II multicenter trial of low‐dose cidofovir for BK polyomavirus nephropathy
Source: Transpl Infect Dis. 2024 Sep 3;26(6):e14367. doi: 10.1111/tid.14367 (PMC11666883; doi:10.1111/tid.14367)

**Supplemental Data.**

**Appendix 1. Patient Enrollment by Study Site**

|  | **Investigator** | **Enrolled** |
| --- | --- | --- |
| University of Alabama at Birmingham | Clifton Kew | 1 |
| University of Chicago | Michelle A. Josephson | 3 |
| University of Colorado at Denver | Alexander C. Wiseman | 4 |
| University of Wisconsin Medical School | Milagros Samanego-Picota | 1 |
| University of Washington Medical Center | Ajit P. Limaye | 3 |
| University of California, San Francisco | Deborah B. Adey | 1 |
| California Pacific Medical Center | V. Ram Peddi | 8 |
| University of Minnesota | Jo-Anne H. Young | 1 |

**Supplemental Figure 1.** Absolute plasma BKPyV loads (log_10_ scale) during study period


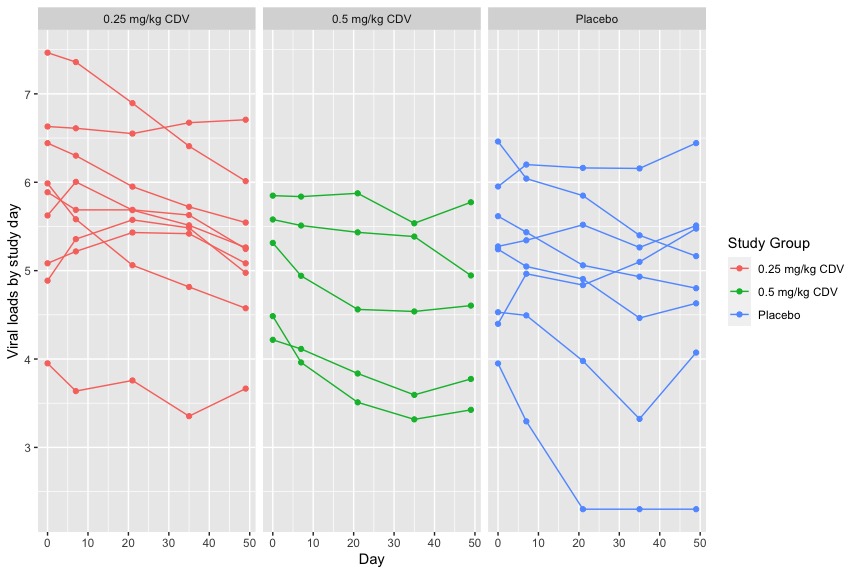


**Supplemental Figure 2.** Absolute urine BKPyV loads (log_10_ scale) during study period


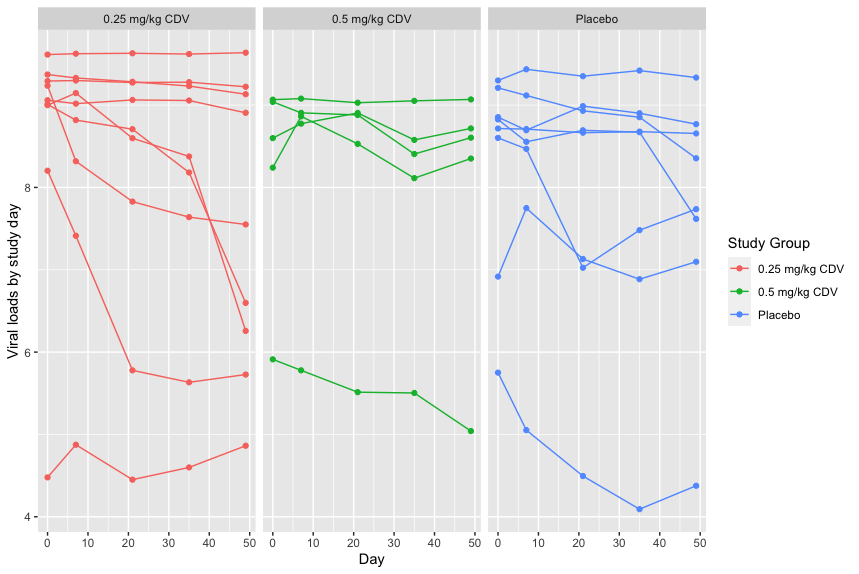


**Supplemental Figure 3.** Absolute plasma BKPyV loads (non-logarithmic scale) during study period


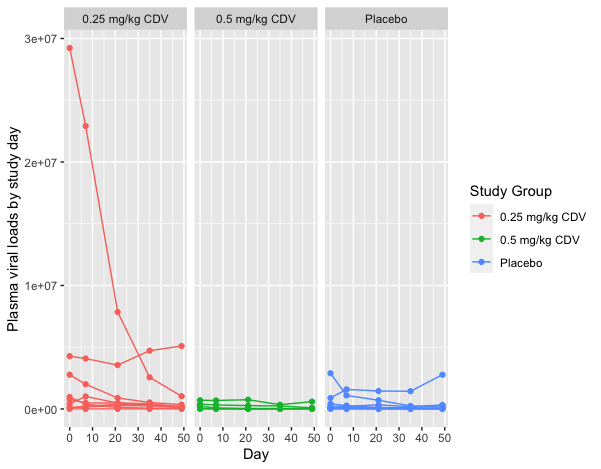


**Supplemental Figure 4.** Absolute urine BKPyV loads (non-logarithmic scale) during study period


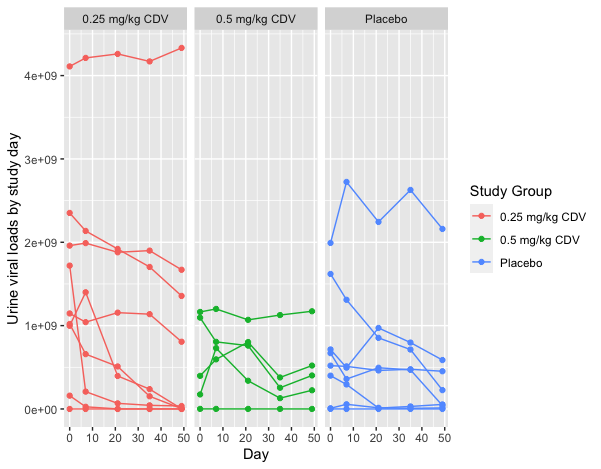

Supplement: Supplementary file 1 — Supporting information [file TID-26-e14367-s002.docx]
