# Supplementary material for: A randomized, placebo‐controlled, dose‐escalation phase I/II multicenter trial of low‐dose cidofovir for BK polyomavirus nephropathy
Source: Transpl Infect Dis. 2024 Sep 3;26(6):e14367. doi: 10.1111/tid.14367 (PMC11666883; doi:10.1111/tid.14367)
Supplement: Supplementary file 2 — Supporting information [file TID-26-e14367-s003.pdf]

A RANDOMIZED, PLACEBO-CONTROLLED, DOSE-ESCALATION STUDY TO ASSESS THE SAFETY AND EFFECT OF CIDOFOVIR IN RENAL TRANSPLANT RECIPIENTS WITH BK VIRUS NEPHROPATHY (CASG 209)

DMID Protocol Number: 04-047

DMID Funding Mechanism: Contract # NO1-AI-30025

Pharmaceutical Support Provided by: Gilead Sciences Inc., Foster City, California

Principal Investigator: Richard J. Whitley, M.D.

DMID Protocol Champion: Walla Dempsey, Ph.D.

DMID Medical Monitor: Steve Heyse, MD

Protocol Chair: Ajit P. Limaye, M.D., University of Washington, Seattle

Protocol Co-Chairs: John W. Gnann, Jr, M.D., University of Alabama at Birmingham
Deb Adey, M.D., University of California, San Francisco

CASG Central Unit Administration: Penelope M. Jester, BSN, MPH, CCRC
Ilet Dale, BSN, MSM

Version Number: 3.0

20 April 2007

Previous versions:
Version 2.0 dated May 6, 2005
Version 1.0 dated February 11, 2005

STATEMENT OF COMPLIANCE

Each investigator must adhere to the protocol as detailed in this document. Each investigator will be responsible for enrolling only those study participants who have met protocol eligibility criteria. This trial will be conducted in compliance with the protocol, International Conference on Harmonization Good Clinical Practice E6 (ICH-GCP) and the following applicable regulatory requirements:

- U.S. Code of Federal Regulators applicable to clinical studies (45 CFR 46 and 21 CFR including parts 50 and 56 concerning informed consent and IRB regulations, if under IND, 21 CFR 312).

- Directive 9115071EEC: The Rules Governing Medicinal Products in the European Community.

- Completion of Human Subjects Protection Training
  Refer to:

  http://grants.nih.gov/grants/guide/notice-files/NOT-OD-01-061.html
  http://cme.cancer.gov/clinicaltrials/learning/humanparticipant-protections.asp

AGREEMENT WITH PROTOCOL

By my signature below, I , ___________________________________, agree to conduct this protocol "**A RANDOMIZED, PLACEBO-CONTROLLED, DOSE-ESCALATION STUDY TO ASSESS THE SAFETY AND EFFECT OF CIDOFOVIR IN RENAL TRANSPLANT RECIPIENTS WITH BK VIRUS NEPHROPATHY"** and the attachments, and provide the necessary assurances that this trial will be conducted according to all stipulations of the protocol, including all statements regarding confidentiality, and according to local legal and regulatory requirements and applicable U.S. clinical sites. I understand that no deviations from the protocol may be made without permission of the CASG. I also understand that information in this protocol is proprietary and therefore, I agree to maintain the confidentiality of the protocol and data generated from the protocol until permission to publicize results is granted by the NIH and the CASG.

SITE PRINCIPAL INVESTIGATOR:

_ _______________________________________ __________________
Signature Date

TABLE OF CONTENTS

STATEMENT OF COMPLIANCE ..... **II**

AGREEMENT WITH PROTOCOL ..... **III**

LIST OF ABBREVIATIONS ..... **VII**

PROTOCOL SUMMARY ..... **X**

1. BACKGROUND **1**

1.1. BK VIRUS AND RENAL NEPHROPATHY 1
1.2. CIDOFOVIR 2
1.3. CLINICAL EXPERIENCE WITH CIDOFOVIR AND BK INDUCED NEPHROPATHY 3
1.4. STUDY AND DOSE RATIONALE 5
1.5. RISKS TO SUBJECTS 8
1.6. STUDY CONDUCT 11

2. OBJECTIVES **13**

2.1. PRIMARY OBJECTIVE 13
2.2. SECONDARY OBJECTIVES 13

3. STUDY DESIGN **14**

3.1. OVERVIEW 14
3.1.1 Guidelines for Reduction in Immunosuppression *14*
3.1.2. DLT Definition. *15*
3.1.3. General Plan *16*
3.2. ENDPOINTS 19
3.2.1. Primary Endpoint *19*
3.2.2. Secondary Endpoints *20*
3.2.3. Tertiary Endpoint *20*

4. STUDY POPULATION **21**

4.1. INCLUSION CRITERIA 21
4.2. EXCLUSION CRITERIA 22
4.3. RANDOMIZATION AND BLINDING 22
4.4. RECRUITMENT 23

5. STUDY PROCEDURES **24**

5.1. CLINICAL EVALUATIONS 24
5.1.1. General Assessments *24*
5.1.2. Laboratory Evaluations *24*
5.1.3. Pharmacokinetic Studies *25*
5.1.3.1. Clinical Pharmacology Plan *25*
5.1.3.2. Cidofovir Pharmacokinetic Sampling - Limited Sample Data Collection *27*
5.1.3.2.1. First dose pharmacokinetics *27*
5.1.3.2.2. Multiple post-last dose pharmacokinetics *27*
5.1.3.2.3. Intensive Sample Data Collection for the MTD *27*
5.1.3.2.4. Urine Data Collection (only for subjects at the MTD Cohort) *27*
5.1.4. Ocular Exam *28*
5.1.5. Renal Biopsy *28*
5.1.6. Assessment of Emergence of Resistance to Cidofovir *29*

5.1.7. Pregnancy Prevention Counseling......29
5.1.8. Specimen Preparation, Handling and Shipping......29
5.1.8.1. Instructions For Specimen Preparation, Handling and Storage.......29
5.1.8.2. Specimen Shipment......30
5.2. SCHEDULE OF PROCEDURES (SEE APPENDIX A)......30
5.2.1. Day: (–) 21 to 0......30
5.2.1.1. Screening period: (-) 21 to Day 0......30
5.2.1.2. Within 48 hours prior to dosing unless otherwise stated.......32
5.2.2. Day 0 (dosing)......33
5.2.3. Day 7 ± 1 (unless otherwise indicated)......34
5.2.4. Day 14 ± 2 (blood may be obtained at remote labs and shipped to local site)......35
5.2.5. Day 21 ± 1 (unless otherwise indicated)......36
5.2.6. Day 28 ± 2 (blood samples may be obtained at remote labs and shipped to the site lab)......37
5.2.7. Day 35 ± 1 (unless otherwise indicated)......37
5.2.8. Day 42 ± 2 (blood samples may be obtained at remote labs and shipped to the site lab)......39
5.2.9. Day 49 ± 3......40
5.2.10. Day 120 ± 14......41
5.3. EARLY TERMINATION DURING WEEKS 1-5......41

6. SUBJECT & STUDY DISCONTINUATION......42

6.1. SUBJECT DISCONTINUATION......42
6.2. STUDY DISCONTINUATION......43

7. STUDY TREATMENT......44

7.1 STUDY PRODUCTS......44
7.1.1 Cidofovir (VISTIDE®)......44
7.1.2. Placebo......44
7.2. PACKAGING AND LABELING......44
7.3. STORAGE AND HANDLING......44
7.3.1. Cidofovir (VISTIDE®)......44
7.3.2. 0.9% Normal Saline......45
7.4. DISPOSAL......45
7.5. DRUG ACCOUNTABILITY......45
7.6. STUDY DRUG PREPARATION AND ADMINISTRATION......45
7.7. DOSE MODIFICATION......46
7.8. WITHDRAWAL OF STUDY DRUG......47
7.9. CONCOMITANT THERAPY......47
7.10. PROHIBITED THERAPIES......47

8.0. ASSESSMENT OF SAFETY......49

8.1. ADVERSE EVENT......49
8.2. SERIOUS ADVERSE EVENT......51
8.3. REPORTING REQUIREMENTS......52
8.3.1. Reporting Adverse Events......52
8.3.2. Reporting Serious Adverse Events......53
8.3.3. Flowchart Summarizing AE and SAE Reporting......55
8.3.4. Pregnancy......56
8.3.5. Expected Adverse Events......56

9.0. STATISTICAL METHODS......57

9.1. GENERAL CONSIDERATIONS......57
9.2. SAMPLE SIZE JUSTIFICATION......58
9.2.1. Safety Evaluation......58
9.2.2. Efficacy Evaluation......59

9.3. Treatment Assignment and Blinding 59
9.4. Safety and Efficacy Analyses 60
9.4.1. Safety Analysis 60
9.4.2. Efficacy Analysis 60
9.4.3. Subject Disposition....61
9.5. Pharmacokinetic and Pharmacodynamic Analyses 61

10.0. STUDY AND INVESTIGATOR REQUIREMENTS....63

10.1. Protocol Adherence 63
10.2. Quality Assurance and Quality Control 64
10.3. Case Report Forms....64
10.4. Source Documentation 64
10.5. Site Monitoring....65
10.6. Data Handling and Record Retention 66
10.7. Informed Consent 66
10.8. IRB/IEC Approval 66
10.9. Anonymity and Confidentiality 67
10.10. Future Use of Specimens 67
10.11. Health Insurance Portability and Accountability Act of 1996 (HIPAA) 68

11.0. REFERENCES 69

APPENDIX A. SCHEDULE OF PROCEDURES 72

LIST OF ABBREVIATIONS

ACTG	AIDS Clinical Trials Group
AE	Adverse Event
ALT	Alanine Aminotransferase
ANC	Absolute Neutrophil Count
AST	Aspartate Aminotransferase
AUC	Area Under the Curve
BP	Blood Pressure
BKVN	BK Virus Nephropathy
C^o	Celsius
C_{max}	Maximum Concentration
CASG	Collaborative Antiviral Study Group
CFR	Code of Federal Regulations
CLcr	Creatinine Clearance
CL_T	Total Clearance
CMV	Cytomegalovirus
CRF	Case Report Form
DLT	Dose Limiting Toxicity
DMID	Division of Microbiology and Infectious Diseases
DNA	Deoxyribonucleic Acid
DSMB	Data and Safety Monitoring Board
FK506	Tacrolimus
FDA	Food and Drug Administration
FWA	Federalwide Assurance
g	Gram
GCP	Good Clinical Practices
GCSF	Granulocyte Colony Stimulating Factor
GFR	Glomerular Filtration Rate
GLS	Generalized Leased Squares
HIPAA	Health Insurance Portability and Accountability Act

HIV	Human Immunodeficiency Virus
HR	Heart Rate
HTLV	Human T-cell Lymphotropic Virus
IB	Investigator Brochure
IC_{50}	Inhibitory Concentration
ICF	Informed Consent Form
ICH	International Conference on Harmonization
ICU	Intensive Care Unit
IEC	Institutional Ethics Committee
IND	Investigational New Drug
INR	International Normalization Ratio
IRB	Institutional Review Board
IS	Immunosuppression
IUD	Intrauterine Device
IV	Intravenous
IVIG	Intravenous Immunoglobulin
JC	JC Virus
LDH	Lactate Dehydrogenase Isoenzymes
kg	Kilograms
mg	milligrams
mL	Milliliters
min	Minute
MMF	Mycophenolate Mofetil
MOP	Manual of Procedures
MRT	Mean Residence Time
MTD	Maximum Tolerated Dose
NCI CTC	National Cancer Institute Common Toxicity Criteria
NIAID	National Institute of Allergy and Infectious Diseases
NSAID	Nonsteroidal Anti-inflammatory Drug
NIH	National Institutes of Health

NS	Normal Saline
OHRP	Office of Human Research Protection
PCR	Polymerase Chain Reaction
PD	Pharmacodynamics
PI	Principal Investigator
PK	Pharmacokinetics
PT	Prothrombin Time
PTT	Partial Thromboplastin Time
QC	Quality Control
QA	Quality Assurance
RBC	Red Blood Cells
RR	Respiratory Rate
SAE	Serious Adverse Event
SMC	Safety Monitoring Committee
SOP	Standard Operating Procedure
T	Temperature
T_{max}	Maximum Time
μg	Microgram
μL	Microliter
USP	United States Pharmacopeia
UTI	Urinary Tract Infection
WBC	White Blood Cells

PROTOCOL SUMMARY

TITLE:
A Randomized, Placebo-Controlled, Dose-Escalation Study to Assess the Safety and Effect of Cidofovir in Renal Transplant Recipients with BK Virus Nephropathy

STUDY PHASE: I/II

STUDY POPULATION: Adult (age 18 and older) male and female renal or renal/pancreas transplant patients who have newly diagnosed BK Virus nephropathy confirmed by positive plasma PCR.

STUDY PARTICPATION: Approximately 8-10 weeks of active participation with a single follow up phone call at 4 months

STUDY DURATION: 3 years

NUMBER OF SITES: 15 - 20, estimated

DESCRIPTION OF AGENT: Cidofovir (VISTIDE®) is a marketed product for treatment of CMV disease (retinitis) in HIV infected patients. It is packaged as a sterile, hypertonic aqueous solution for intravenous infusion only. The control for this study is sterile, 0.9% normal saline for intravenous use.

OBJECTIVES:

Primary

- To evaluate the safety and tolerability of three dose levels of cidofovir when administered to renal transplant recipients with BK virus nephropathy
- To identify the maximum tolerated doses (MTD) among the three dose levels of cidofovir in renal transplant recipients with BK virus nephropathy

Secondary

- To evaluate the antiviral effect of cidofovir at each of three dose levels
- To evaluate the pharmacokinetics of cidofovir in renal transplant recipients with underlying renal impairment
- To evaluate the pharmacodynamics of cidofovir in this setting
- To evaluate allograft function at the completion of the study
- To assess allograft rejection at the completion of the study

STUDY DESIGN:

This is a randomized, double-blind, placebo-controlled, sequential dose escalation study to evaluate the safety, tolerability, MTD, PK/PD and preliminary efficacy of cidofovir in kidney transplant recipients with newly diagnosed BKVN. Patients with BKVN diagnosed by positive plasma PCR or renal allograft biopsy will be randomized to receive study drug within 60 days of the date of the renal biopsy or plasma PCR assay that established the diagnosis of BKVN. The study consists of three dose cohorts (0.25 mg/kg, 0.5 mg/kg and 1.0 mg/kg); each cohort will consist of approximately 12 subjects randomized 2:1 to receive either cidofovir or placebo (0.9% normal saline) to define the maximum tolerated dose (MTD) among the three specified doses of cidofovir. Once the MTD is established, approximately 12 additional patients (randomized 2:1) will be enrolled at that dose. The MTD is defined as the dose in which no more than 2 of the 8 cidofovir treated subjects experience a dose limiting toxicity (DLT), as defined below. The target enrollment is 48 subjects if all dose cohorts are fully enrolled. A 25% over-enrollment may be tolerated to allow for continued enrollment of subjects in the lower dose cohort if data are under concomitant review by the Data and Safety Monitoring Board or to replace non-evaluable study participants. Study participants who have been randomized and have received cidofovir/placebo (in any cohort) will be considered non-evaluable if they discontinue from the study or die for any reason except toxicities definitely related to study treatment, including DLTs. These subjects may be replaced.

Cohort	Dose	Cidofovir (n)	Placebo (n)
**I**	**0.25 mg/kg**	**8**	**4**
**II**	**0.5 mg/kg**	**8**	**4**
**III**	**1.0 mg/kg**	**8**	**4**
**MTD**	**X (to be determined)**	**8**	**4**
		**32**	**16**

All subjects will have immunosuppression reduced (as recommended in the "Guidelines for Reduction in Immunosuppression" in Section 3.1.1.) then be randomized 2:1 to either IV cidofovir or placebo (0.9% normal saline). There will be a 5 week drug administration period (4 doses) followed by a 2 week end-of-study observation and evaluation period for each cohort At about 3 months after last dose of study infusion, a member of the research staff will assess the study participant and counsel on pregnancy status via a phone call.

For the purposes of this study, DLT's are defined as:
1) > 50% decrease in glomerular filtration rate (GFR) from baseline (GFR within 48 hours prior to first study infusion)
2) ANC < 500 per microliter despite administration of GCSF (Granulocyte Colony Stimulating Factor). This study will be overseen by a Data and Safety Monitoring Board (DSMB) who will review the data after each dose cohort is completed. Enrollment will not stop while the DSMB reviews the data. Enrollment will proceed to the next dose cohort during the DSMB review if no more than 2 subjects experiencing DLTs are observed in a cohort. If a 3rd subject experiences a DLT occurs in any dose cohort, further new enrollments at that dose will cease, but subjects who are already enrolled at that dose will continue receiving study medication. Any additional eligible subjects for the study will continue to be enrolled, but only at the prior dose as determined by the DSMB to be safe. With the development of a 3rd study subject experiencing a DLT in any cohort, the DSMB will convene and determine whether continued enrollment at that dose is acceptable. If the DSMB determines that continued enrollment at that dose is appropriate,

then any new eligible subjects will be enrolled at that dose until that dose cohort is fully accrued. Because the study is blinded, only the DSMB will be able to assess DLTs according to randomization arm (cidofovir versus placebo).

In addition to safety assessments, the pharmacokinetics of cidofovir and effect of treatment on virologic endpoints will be assessed.

NUMBER OF SUBJECTS:

It is anticipated that a maximum of 48 subjects will be enrolled in this study, if all cohorts are fully enrolled. Approximately 12 subjects in Cohorts I, II and III will be randomized in a 2:1 ratio to receive cidofovir or placebo, respectively. Approximately 12 additional subjects (8 cidofovir, 4 placebo) will be enrolled at the MTD (MTD cohort). A 25% over-enrollment may be tolerated to allow for continued enrollment of subjects in a lower dose cohort if data are under review by the DSMB or to replace non-evaluable subjects.

STUDY INCLUSION AND EXCLUSION CRITERIA:

Inclusion Criteria

- Aged ≥ 18 years
- Kidney or kidney/pancreas transplant recipient
- New onset BKVN diagnosed by a positive plasma PCR assay for BK virus DNA or by a renal biopsy demonstrating BK virus (by immunohistochemistry, electron microscopy and/or in situ hybridization) obtained as part of standard medical care within 60 days prior to receipt of first dose of study drug.
- BK virus load in plasma > 10,000 copies/mL within prior 21 days.
- Glomerular filtration rate > 30 mL/min using Levey calculations (see Section 5.1.3.1.)
- Absolute neutrophil count >1000/µl (with GCSF support as necessary)
- Women must be post-menopausal, surgically sterile or willing to use adequate contraception (barrier method with spermicide, IUD, oral contraceptives, implant or other licensed hormone method) from time of study enrollment through 1 month after the last dose of study treatment.

Men must be surgically sterile or willing to use contraception (barrier method with spermicide) from time of study enrollment through 3 months after the last dose of study treatment.

Exclusion Criteria

- Unable to obtain valid informed consent
- History of intolerance to cidofovir or related compounds (i.e. other nucleotide derivatives [adefovir or tenofovir])
- Pregnant or breast feeding women
- Prior treatment with cidofovir within the last 2 weeks
- Receipt of another investigational drug with proven nephrotoxic drug interaction with cidofovir or known antipolyoma virus activity one month prior to study entry
- Contraindication to renal biopsy (e.g., anticoagulant medication, unwilling to undergo biopsy)
- Currently receiving or anticipated to receive any of the following within 2 weeks of randomization:
  - Amphotericin preparation (intravenous)
  - Aminoglycosides (intravenous)
  - Platinum – based chemotherapeutic agents
  - NSAIDs – non steroidal anti-inflammatory drugs (aspirin given for cardioprotective treatment is acceptable up to 650 mg po daily)
  - Foscarnet
  - Pentamidine (intravenous)
  - Probenecid
  - Leflunomide
- Hypotony or uveitis

SAFETY EVALUATIONS:

Physical examination, including vital signs, clinical laboratory assessments (including BK viral load in blood/urine), and collection of adverse event data will be performed serially. Renal biopsy will be performed in any subject who develops > 50% reduction in glomerular filtration rate. In addition, ocular symptoms and ANC will be monitored throughout the study. Within 48 hours prior to administration of each dose of study drug, safety laboratory assessments in serum and urine will be done.

EFFICACY EVALUATIONS:

The effect of cidofovir on BK virus will be determined by:

- percentage of subjects who achieve an undetectable BK virus urine and plasma PCR between baseline and end of treatment;
- rate of reduction in urine and plasma BK virus load by quantitative PCR between baseline and end of treatment;
- time to reduction in BK virus urine and plasma PCR.

PHARMACOKINETIC EVALUATIONS:

Pharmacokinetic (PK) determinations of cidofovir will be evaluated in all subjects enrolled in each dose cohort and at the MTD, and will include $AUC_{0-6h,}$ C_{max}, T_{max}, MRT. A more intensive PK sampling of blood and urine will be assessed in subjects in the MTD cohort. Cidofovir pharmacokinetic parameters will be correlated with the observed adverse events and anti-BK virus activity.

PHARMACODYNAMIC EVALUATIONS:

The relationship between cidofovir exposure and anti-BK virus activity will be evaluated by correlating the rate and extent of reduction in BK virus DNA with the pharmacokinetic parameters of cidofovir in blood and urine.

Protocol Summary – continued

Schematic A of Study Design BK Flowchart Screening for All Cohorts

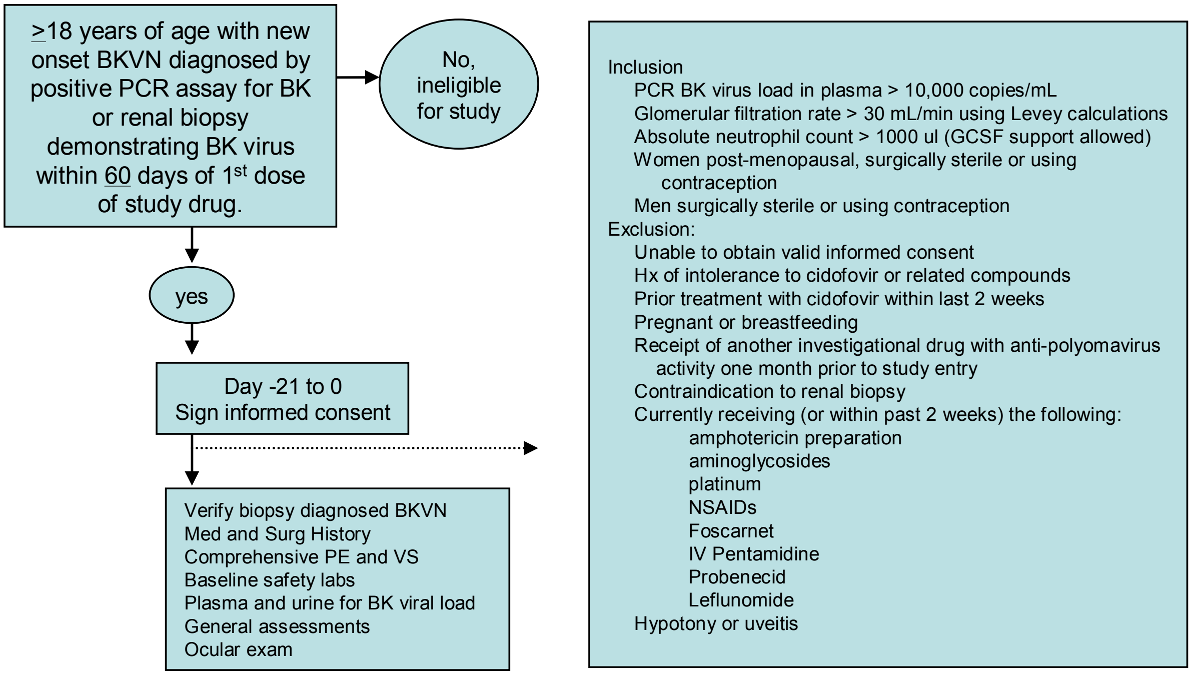

Schematic B of Study Design BK Flowchart for Cohorts 1, 2, and 3 from Baseline to End of Study

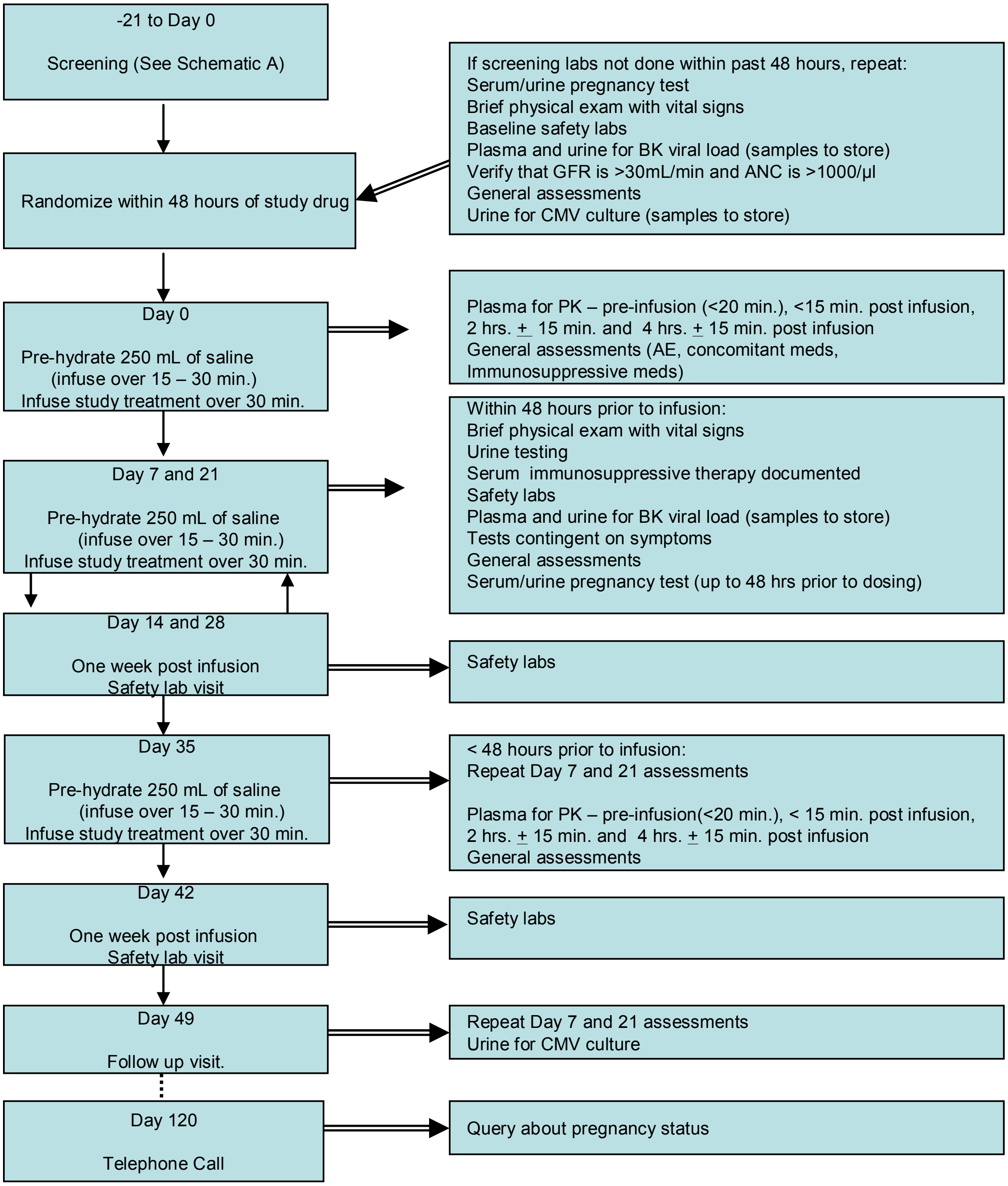

Schematic C of Study Design BK Flowchart for Cohorts MTD From Baseline to End of Study

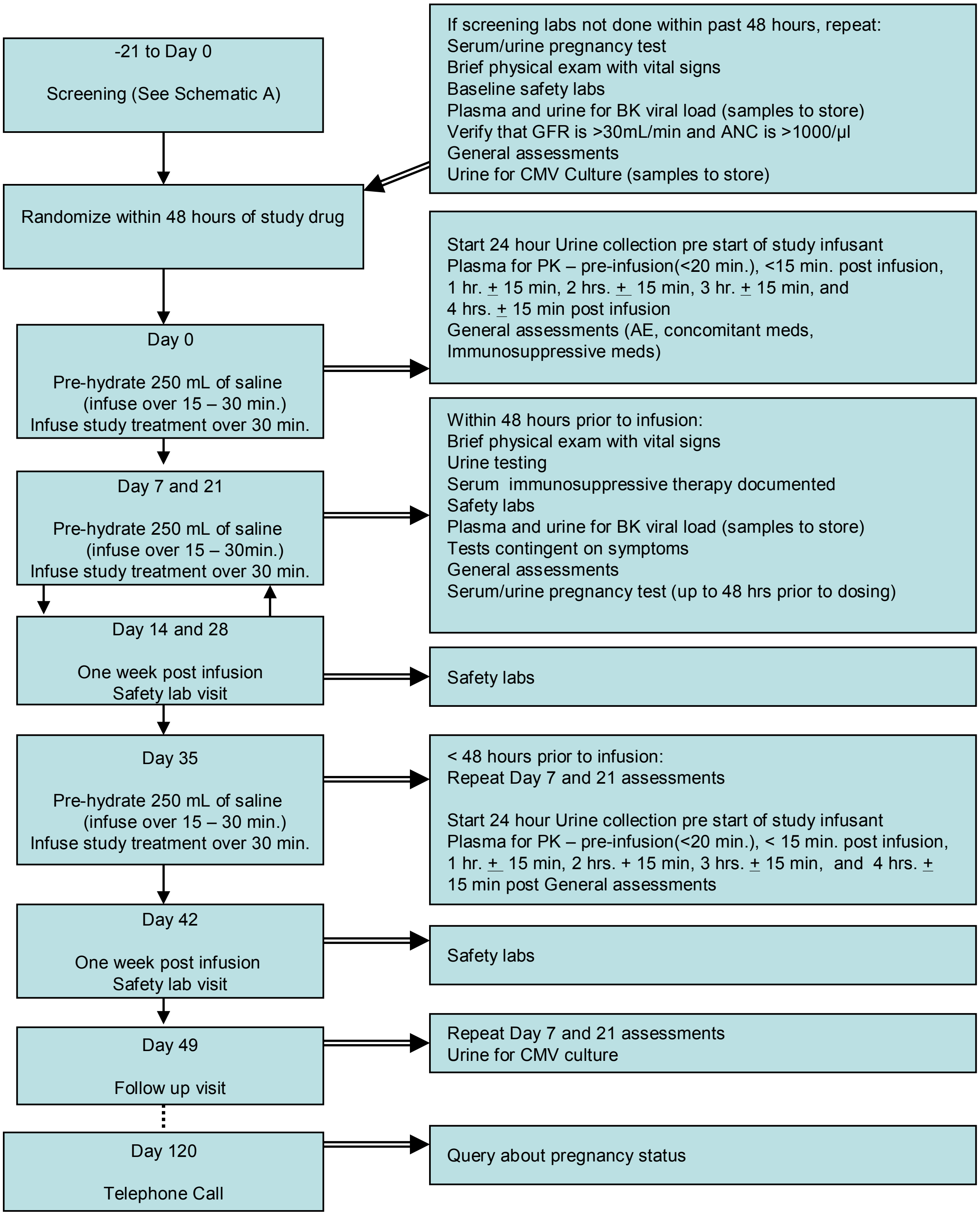

1. BACKGROUND

1.1. BK Virus and Renal Nephropathy

The human polyomaviruses include JC and BK virus. Both are small, non-enveloped, 45-55 nm in size, and contain a double-stranded DNA genome of approximately 5,000 base pairs. Seroepidemiologic studies have demonstrated that BK virus infection is almost universal, and occurs in early childhood.[1] Following initial infection (presumably through the respiratory route), BK virus appears to preferentially establish latency in the genitourinary tract.[2] BK virus rarely reactivates in immunocompetent adults.[3] However, in the setting of immunosuppression (such as kidney transplantation), asymptomatic BK virus infection can be readily detected in urine.[4,5,6] Rarely, BK virus is associated with symptomatic infection, and the clinical manifestations appear to vary according to the specific clinical setting. In kidney transplant recipients, ureteral stenosis and kidney infection (BK virus associated nephropathy/nephritis [BKVN]) are the most commonly described clinical manifestations of symptomatic BK virus infection.

Several studies have documented an increasing incidence of BK virus-associated nephropathy [BKVN] in kidney transplant recipients.[7,8,9,10,11] Although the incidence of BKVN has not been precisely defined, a recent prospective study has demonstrated an incidence of BKVN of 8% in kidney transplant recipients who received 1 or more of the newer immunosuppressive agents, such as tacrolimus (FK506), mycophenolate mofetil (MMF), and sirolimus (Rapamune).[6,12] Other groups have reported incidences of BKVN ranging from 1 to 5%.[13,14] The precise factors leading to an increased incidence are unknown, but an association with the newer (more potent) immunosuppressive agents has been noted by several investigators.[13,14,11] Two specific lines of evidence suggest that BKVN may be a consequence of “over-immunosuppression”. First, BKVN has been seen much more commonly in the era of more potent immunosuppressive agents (i.e. “post-cyclosporine era”), and most patients with documented BKVN have received one or more of these newer immunosuppressive agents. Second, some patients with BKVN appear to have clearance of the virus from blood and kidney after reduction in immunosuppression drug regimen.[6,15,16]

Progression to BKVN often occurs in the absence of most clinical signs with the exception of an increase in creatinine over several weeks in many cases. Currently, histopathologic examination of the kidney allograft is the "gold standard" for the definitive diagnosis of BKVN. However, several investigators have shown that BK virus DNA can be detected in blood (by PCR) in virtually all patients at the time of histological diagnosis of BKVN.[16,15,6] Furthermore, BK virus DNA can be detected in blood or urine for up to several months before renal dysfunction or histologic evidence of BKVN (as detected by renal biopsy) develops.[15,6,16] Thus, if a safe and efficacious means of intervention were identified, a preemptive approach could be a very useful strategy to potentially prevent the development of clinically manifest BKVN (i.e. reactivated BK virus infection could be prevented from progressing to invasive disease). Recently, quantitative assays for BK virus DNA (PCR) have been developed and reported.[17,16] Importantly, BK virus load in blood has been shown to correlate with both renal function and histologic evidence of BK virus in the allograft kidney.[16,18,6,15] Thus, quantitative molecular assays provide a useful non-invasive means by which to assess responses to interventions such as reduction in immunosuppression or antiviral therapy.

The current approach to patients with BKVN is to reduce the immunosuppression regimen (based on the consensus opinion that BKVN may result from "over-immunosuppression"). However, despite reduction in immunosuppression, up to 30-70% of patients have progressive deterioration in renal function eventually resulting in allograft loss.[19,11,16,20] The progressive deterioration in renal function typically occurs over months, and is thought to be due to a combination of persistent viral infection, rejection, (induced by reduction in immunosuppression and/or virally-mediated) and fibrosis.[21,11,20] Thus, the current management strategy is unsatisfactory, and associated with a poor allograft outcome in a significant proportion of patients.

1.2. Cidofovir

Cidofovir is an acyclic nucleotide analog of cytosine. The parent nucleotide is converted via cellular enzymes to the pharmacologically active diphosphate anabolite, which is highly selective because of its greater affinity for viral DNA polymerase than for human DNA polymerase. Cidofovir is licensed for the treatment of CMV retinitis in patients with HIV infection at a dose of

5 mg/kg IV. Cidofovir has demonstrated *in vivo* and/or *in vitro* activity against a broad spectrum of DNA viruses including herpes viruses, poxviruses, and adenovirus. Recently, cidofovir has been shown to exhibit *in vitro* activity against murine polyomavirus strains, with IC_{50}'s ranging from 4-7 µg/mL.[22] In addition, Farasati et al have reported in vitro activity of cidofovir against human strain of BKVN.[22.b.]

1.3. Clinical experience with cidofovir and BK induced nephropathy

In addition to the activity of cidofovir against polyomavirus strains *in vitro*, there are several anecdotal reports of clearance of BK virus from both the blood and allograft of kidney transplant recipients who were treated with low-dose cidofovir and reduction in immunosuppression [summarized in Table 1]. In general, doses between 0.25 mg/kg to 1.0 mg/kg were administered over several weeks, in most cases, without concomitant probenecid. All of these studies were uncontrolled and non-standardized, but do suggest that low doses of cidofovir were safely tolerated in many patients even with underlying renal dysfunction and concomitant nephrotoxic medications. In most cases cidofovir was well tolerated, even though patients had increased creatinine levels prior to treatment. Although not published, anecdotal reports from the University of Pittsburg suggest that doses of 3.0 mg/kg or higher may be associated with renal toxicity and may not be well tolerated in this patient population. The administration of cidofovir to renal transplant patients with BKVN has increased in transplant centers as the result of these publications, even though no controlled studies have formally demonstrated either the safety or the efficacy or the dose/duration of treatment. Conservatively, one can estimate that over 100 patients have received various doses and regimens of cidofovir to treat BKVN. From the published data and the experience of the protocol chairs alone, at least 85 kidney transplant recipients have received cidofovir for treatment of BKVN.

Table 1.

Author	Year	Publication	# Patients	Immunosuppression (IS)	Cidofovir Dose	Duration	Creatinine Initial/Final	Response	Comment
Bjorang[23]	2002	Nephro, Dialy & Transplantation	Case report	Prednisone reduced, mycophenolate decreased	0.25 mg/kg IV x1, 0.42 mg/kg IV x 11 doses over 7 months		3.5 mg/dl 3.4 mg/dl at completion	Cleared blood of virus, urine persistently positive	Diagnosed 22 months post transplant
Scantlebury[24]	2002	Graft	16 (1 pediatric, 15 adult)	Decrease tacrolimus, decrease or stop steroids, sirolimus and/or mycophenolate markedly decreased or ceased	0.20 – 1.0 mg/kg IV q 1-4 weeks,			25 % lost the allograft, 75 % maintained graft function (11/12 cleared virus in the plasma)	Diagnosed 3-73 months post transplant, 10 by tissue, 6 by plasma PCR
Kadambi[18]	2003	Am J Transplant	Case series, n=2	Decrease IS: MMF decreased by 50% - 75%, tacrolimus decreased by 29% - 50%	2.5 mg/kg (with po probenecid in patients #1) Q 2 weeks		#1 3.3 mg/dl / 2.4 mg/dl<br><br>#2 2.7 mg/dl/ 1.5 mg/dl		Plasma cleared of virus, persistent positive urine
Keller[25]	2003	Nephro, Dialy & Transplantation	Case Report, n=1	Tacrolimus reduced by 37.5%, and sirolimus discontinued, mycophenolate mofetil added	0.25 mg/kg IV Q 2 weeks	8 doses	2.0 mg/dl 1.8 mg/dl with decrease IS, 1.6 mg/dl with cidofovir	Histologic clearance of BK, urine remained positive	Dx at 8 months post transplant, all by tissue biopsy
Vats[17]	2003	Transplantation	Case series, n=4		0.25 –1.0 mg/sl without probenecid, 1 patients 3.0 mg/kg with probenecid	1-4 doses	1.5 – 2.9 mg/dl at DX;<br>3.0-7.2 at peak;<br>1.7 – 3.2 mg/dl at publication		Dx 9-11 months post transplant
Vats[26]	2003	Abstract ATC 2003	26 (4 pediatric, 22 adult	Reduction in IS	0.25 – 1.0 mg/kg IV Q 2-4 weeks without probenecid	Followed 3-42 weeks		4 (15%) lost allograft, 50 % improved creatinine, viruria decreased by 4-6 logs	IS reduction resulted in decrease in viral load, but cidofovir resulted in a faster reduction in viral load
Herman[27]	2003	Abstract ATC 2003	1, pediatric case report	Reduction in IS, but increased methyprednisilone	1.0 mg/kg Q 2 weeks with probenecid	Not stated	Stabilized, but elevated	Decreased plasma viral load, persistent viruria	

1.4. Study and Dose Rationale

Given the limitations in the current strategy (reduction in immunosuppression) for the management of patients with BKVN, new approaches are required. As stated above, several lines of evidence suggest that cidofovir may have activity against BK virus, and might be clinically useful for the treatment of BKVN in kidney transplant recipients. First, cidofovir demonstrates *in vitro* activity against murine and simian polyomavirus strains[22, 28] and appears to have activity against a related human polyomavirus (JC virus) *in vivo*[29] and a human BK virus strain.[22b] Second, there are several reports of favorable responses to cidofovir therapy (when combined with reduction in immunosuppression) in kidney transplant recipients with BKVN [Table 1]. Several important limitations of these studies should be noted. They were uncontrolled, used varying doses of cidofovir, and used non-standardized definitions of clinical/virologic responses. However, these preliminary data suggest that low-dose cidofovir might potentially be safely tolerated in kidney transplant recipients, and that there may be a clinical/virologic benefit of cidofovir therapy in the treatment of BKVN in this clinical setting. In addition, with the advent of quantitative molecular assays to monitor BK virus load in blood and urine (and their demonstrated correlation with clinical response and BK virus load in kidney), specific tools are now available with which to assess and quantify responses to antiviral therapy.

The doses and dosing regimens to be evaluated in this trial are 0.25, 0.5 and 1.0 mg/kg of cidofovir given 4 times over a 5-week period. The first two doses will be administered one week apart and the last two doses will be administered 2 weeks apart. The rationale for these doses and the proposed dosing regimen are summarized below.

Typical peak (C_{max}) cidofovir plasma concentrations following a 1.0 mg/kg dose are in the range of 3 µg/mL.[30, 31] Although the doses of cidofovir proposed in this study will likely produce peak (and trough) plasma concentrations below the *in vitro* murine IC_{50} susceptibility values, several lines of evidence suggest that such low doses might be adequate for treatment of BK virus-associated nephropathy (BKVN). First, as noted above, BKVN is an infection that is essentially confined to the genitourinary tract, and specifically, to the tubular epithelium of the kidney. Cidofovir is eliminated from the body almost exclusively by the kidney,[30] and thus would be

predicted to achieve significantly higher concentrations in the urine and genitourinary tract (the primary site of BK virus infection) than in plasma. In addition, animal studies[32, 33] have demonstrated that, following multiple doses, cidofovir accumulates and is highly concentrated in the kidney epithelium (>1000 fold higher than plasma levels). Thus, the concentrations of cidofovir at the primary site of infection (the kidney and genitourinary tract) would be expected to significantly exceed the *in vitro* IC_{50}'s of cidofovir against polyomavirus, even at the low doses to be used in the present study.

Because it is desirable to concentrate cidofovir at the site of the viral infection, and the pharmacokinetic parameters of cidofovir are not substantially altered by the addition of probenecid at doses of cidofovir below 3 mg/kg,[30] concomitant probenecid will not be administered to patients in this study (the maximum dose of cidofovir in the study is 1.0 mg/kg). This approach is supported clinically. In the published studies suggesting possible benefit of cidofovir therapy of BKVN in kidney transplant recipients, cidofovir was typically administered without concomitant probenecid. For these reasons, we will administer cidofovir without concomitant probenecid.

The schedule of doses in this study is 2 doses one week apart followed by 2 doses two weeks apart. This schedule is similar to that used for CMV retinitis. Cidofovir exhibits a dose and schedule-dependent nephrotoxic effect. Concentrations of cidofovir in the kidney at 24 hours post-dose are approximately 100-fold higher than in other tissues (without concomitant probenecid). The accumulation of cidofovir in the renal cortex is apparently the consequence of active tubular secretion into proximal tubular cells and a slower rate of efflux into the tubular lumen. The localization and persistence of cidofovir in the kidney is consistent with the results of animal toxicity studies. Since cidofovir accumulates in the kidney, its toxicity is likely related to the dosing schedule. Animals (guinea pigs) received 25 mg/kg as a single dose, two 12.5 mg/kg doses, four 6.25 mg/kg doses, or five 5 mg/kg doses. The latter schedule induced marked nephrotoxicity (>90% tubules) whereas the single dose resulted in minimal toxicity (<5% tubules). The intermediary dosing regimens were associated with moderate toxicity. These animal data suggest that cidofovir renal toxicity may be reduced through less frequent dosing.

Three phase I/II studies (GS-92-101, 102, and 103) successfully demonstrated the dose-dependent anti-CMV activity, and the temporal development of toxicity permitted the identification of the currently used dose (5 mg/kg once weekly followed by 3-5 mg/kg every other week). Based on these early dose-finding data, a similar dosing schedule strategy to that currently approved for the treatment of CMV retinitis has been chosen. The primary consideration is patient safety through the prevention of nephrotoxicity.

The package insert for cidofovir recommends that patients be pre-hydrated with 1 liter of saline prior to the administration of cidofovir. In this study, the volume of fluids used for pre-hydration will be reduced for several reasons. First, some of the study subjects will have underlying cardiac or renal disease that could predispose them to volume overload. Thus, ensuring that patients receive adequate hydration to minimize any cidofovir-related nephrotoxicity, but not so much as to potentially precipitate volume overload/congestive heart failure, will be important. Because this is a placebo controlled trial, the potential risks of excessive hydration without the potential benefit of receipt of therapy warrants the use of a lower fluid volume. Additionally, since the doses of cidofovir being used in the present study range from 1/20th to 1/5th of the standard dose of 5 mg/kg used to treat CMV retinitis, it seems reasonable to assume that a reduction in infusion volume should still provide some renal protective effects.

In summary, preclinical data suggest activity of cidofovir against BK virus. While published and anecdotal clinical data suggest benefit to the patients, no controlled studies have formally examined the safety and effectiveness of cidofovir in renal transplant patients with BKVN. Since cidofovir has the potential to cause significant nephrotoxicity (at least at the doses used to treat systemic CMV disease) and has not been rigorously studied in kidney transplant recipients who are receiving other nephrotoxic medications (i.e. calcineurin inhibitors), a prospective, protocol-based assessment of cidofovir is warranted. Furthermore, the appropriate dose, pharmacokinetics, safety and tolerability, and antiviral activity of cidofovir against BK virus have not been formally evaluated. Thus, the aims of the present study are:

- To evaluate the safety and tolerability of three dose levels of cidofovir when administered to renal transplant recipients with BK virus nephropathy;
- To identify the maximum tolerated doses (MTD) among the three dose levels of cidofovir in renal transplant recipients with BK virus nephropathy;
- To evaluate the antiviral effect of cidofovir at each of three dose levels;
- To evaluate the pharmacokinetics of cidofovir in renal transplant recipients with underlying renal impairment;
- To evaluate the pharmacodynamics of cidofovir in this setting;
- To evaluate allograft function at the completion of the study;
- To assess allograft rejection at the completion of the study.

The results of this study should provide important background data for the design of future formal efficacy studies of cidofovir. If this study demonstrates that cidofovir is well-tolerated and appears to have antiviral activity, future studies utilizing different durations of therapy, comparison of reduction of immunosuppression versus antiviral therapy, or preemptive therapy could be performed. Furthermore, even if cidofovir was found not to be well-tolerated and/or ineffective, the natural history data obtained from the placebo group (viral load kinetics, biologic variability, blood vs. urine viral loads) would provide very useful background information for the assessment of future antiviral agents. In addition, the off-label use of this relatively expensive, potentially toxic agent could be reduced, and efforts re-directed towards different antiviral (or other) strategies, in the event that cidofovir was found not to be safely tolerated and/or does not demonstrate statistically significant antiviral efficacy in BKVN.

1.5. Risks to Subjects

Nephrotoxicity is the major toxicity associated with use of cidofovir, and cases of acute renal failure resulting in dialysis and contributing to death have occurred in some patients following as few as one or two doses. Nephrotoxicity has usually been associated with doses of 3 mg/kg or higher. In the published reports using lower doses of cidofovir in patients with BKVN, cidofovir was safely tolerated even though the patients had underlying renal dysfunction and concomitant nephrotoxic medications. Renal function (serum creatinine and estimation of glomerular filtration

rate [GFR]) and surrogate markers for proximal tubule injury (proteinuria, glycosuria) will be serially monitored during the course of the study. Subjects who develop a significant reduction in GFR will undergo renal biopsy as part of routine clinical care, and all biopsies will be examined by an expert nephropathologist, in an attempt to define the etiology (e.g., rejection versus progressive BKVN or other medical conditions). Any patient who develops a greater than 50% reduction in GFR will have study drug discontinued; this threshold has specifically been defined as a dose limiting toxicity for the purposes of the study.

In addition to nephrotoxicity, ocular toxicity, including hypotony and uveitis, and neutropenia have been reported in cidofovir recipients, primarily in HIV-infected patients receiving standard doses of cidofovir (5 mg/kg) for CMV retinitis. In the registrational trials, a subset of patients was monitored for intraocular pressure changes; a decrease from baseline pressure of ≥ 50% was reported in 17 of 70 (24%) of patients receiving 5mg/kg maintenance therapy. Severe hypotony (intraocular pressure of 0-1 mm Hg) was reported in 3 patients. Uveitis or iritis has been reported in clinical trials and during Phase IV studies in 15 of 135 (11%) of patients receiving 5 mg/kg maintenance dosing. A single case report has also documented cidofovir-associated uveitis in an HIV-infected patient who reportedly had systemic CMV infection, but not retinitis (although subclinical retinitis accompanying CMV encephalitis was possible).[34] Experience with cidofovir-associated ocular toxicity in non-HIV infected patients is summarized in Table 2. Ocular toxicity was reported in 3 of 131 (2.3%) of human stem cell transplant recipients receiving doses ranging from 3 to 5 mg/kg. In contrast, 0 of 85 (0%) of kidney transplant recipients receiving cidofovir at doses ranging from 0.25 mg/kg to 1.0 mg/kg developed symptomatic ocular toxicity. Thus, it appears that symptomatic ocular toxicity at the doses to be studied in this population will be uncommon. Regardless, as part of routine safety evaluations, as recommended in the cidofovir package insert, monitoring for ocular symptoms, measurement of ocular pressure, and slit lamp examination will be performed by an Eye Care Professional at baseline and end of study.

Table 2. Reports of cidofovir-associated ocular toxicity in non-HIV infected persons.

Standard dose cidofovir in hematopoietic cell transplant recipients

Reference	N	Indication	Dosage	Symptomatic Ocular Toxicity
Ljungman et al.[35] [BMT 2003]	45	Adenovirus	3 to 5 mg/kg (1mg/kg in 1 pt)	0/45 (0%)
Ljungman P et al.[36] [Blood 2001]	82	CMV	3 to 5 mg/kg (1mg/kg in 1 pt)	2/82 (2.7%)
Chakrabarti S et al.[37] [BMT 2001]	4	CMV	5 mg//kg	1/4 (25)

Low-dose cidofovir in kidney transplant recipients with BKVN

Reference	N	Indication	Dosage	Symptomatic Ocular Toxicity
Limaye AP [unpublished data]	11	BKVN	0.25 mg/kg	0/11 (0%)
Adey D [unpublished data]	23	BKVN	0.25 to 0.5 mg/kg	0/23 (0%)
Summary from Table 1	51	BKVN	0.1 to 1.0 mg/kg	0/51 (0%)

Neutropenia has also been described as a toxicity of cidofovir. Since many of the patients in the study will be expected to be on other immunosuppressive agents (mycophenolate mofetil, trimethoprim-sulfamethoxazole, ganciclovir), careful monitoring of hematologic parameters (including ANC) will be performed. Neutropenia occurred in 24% of the patients who received the cidofovir maintenance dose of 5mg/kg. Granulocyte colony stimulating factor was used in 39% of the patients. In this study, subjects will be monitored for changes in white blood cell counts.

Additional adverse events associated with administration of cidofovir include metabolic acidosis, proteinuria, decreased serum bicarbonate, creatinine elevation, and nausea and vomiting.

Cidofovir is carcinogenic, teratogenic and caused hypospermia in animal studies. One 26-week rat subcutaneous toxicology study was stopped at 19 weeks as a result of the induction of palpable masses in female rats which were diagnosed as mammary adenocarcinomas as early as after 6 doses. Cidofovir should be considered a potential carcinogen in humans.

Cidofovir is a pregnancy category C drug and caused hypospermia and was embryotoxic and maternally toxic in rats and rabbits. Cidofovir was mutagenic in the mouse micronucleus test and induced chromosomal aberrations in human peripheral blood lymphocytes in vitro. It is not known whether cidofovir is excreted in human milk.

Cidofovir should not be administered concomitantly with other drugs that have significant nephrotoxic potential, such as: systemically administered aminoglycosides, amphotericin preparations, or pentamidine; platinum based chemotherapeutic agents; foscarnet; or NSAIDs. Such drugs should be discontinued at least 14 days prior to starting cidofovir.

Emergence of resistance to cidofovir in cytomegalovirus (especially with use of low doses) is also a theoretical risk in this study of kidney transplant recipients. Although cidofovir is rarely used to treat CMV infection or disease in kidney transplant recipients (because of the nephrotoxicity at the doses required to treat CMV), we will monitor for this possibility in a population that has a high rate of CMV infection and disease. Since mutations conferring cidofovir-resistance have been well-defined within the UL54 region of the CMV genome[38]and because breakpoints for determining phenotypic cidofovir resistance are available[39], we will use a combination of phenotypic and genotypic methods to assess CMV isolates for possible cidofovir resistance. Pre- and post-study drug urine CMV isolates will be analyzed for resistance.

1.6. Study Conduct

Each investigator must adhere to this protocol as outlined. However, a physician may implement a deviation from, or a change in the protocol to eliminate immediate hazard(s) to trial subjects without prior CASG, NIAID/DMID and/or IRB/IEC approval. Any deviation from the

requirements/procedures outlined in the protocol is considered a protocol deviation and must be documented. Thus, the following are considered to be protocol deviations.

- Failure of either the study participant or investigator to adhere to protocol requirements, study procedures, or study schedules.
- Non-adherence to GCP
- Non-adherence to the protocol-specific Manual of Procedures (MOP)

If a protocol deviation increases the risk to the study participant, the protocol deviation should be reported within 2 business days of identification. All other protocol deviations should be reported within 5 business days of identification. A protocol deviation form must be completed to document the deviation or change and the reason(s) for the deviation. The protocol deviation form should include:

- Nature of the error
- Standard reporting information (subject’s clinical status before and after the event)
- Steps taken to investigate the error
- Steps taken to assure the error will not occur again

The protocol deviation form must be submitted

- To the CASG Central Unit
- To the local IRB/IEC for review and approval/favorable decision (per local IRB-IEC guidelines)
- To the NIAID/DMID for agreement (submitted by the CASG Central Unit) and if required
- To the regulatory authority(ies) (submitted by the CASG Central Unit)

Protocol deviations that result in an adverse event must be reported on a Serious Adverse Event and/or Adverse Event form. All protocol deviations must be reported to the CASG Central Unit as described in the DMID “Source Document Standards” (most current version). The CASG Central Unit will transmit the deviation upon receipt to the NIAID/DMID for documentation.

2. OBJECTIVES

2.1. Primary Objective

- To evaluate the safety and tolerability of three dose levels of cidofovir when administered to renal (or renal/pancreas) transplant recipients with BK virus nephropathy
- To identify the maximum tolerated doses (MTD) among the three dose levels of cidofovir in renal transplant recipients with BK virus nephropathy

2.2. Secondary Objectives

- To evaluate the antiviral effect of cidofovir at each of three dose levels
- To evaluate the pharmacokinetics of cidofovir in renal transplant recipients with underlying renal impairment
- To evaluate the pharmacodynamics of cidofovir in this setting
- To evaluate allograft function at the completion of the study
- To assess allograft rejection at the completion of the study

3. STUDY DESIGN

3.1. Overview

This is a randomized, double-blind, placebo-controlled, sequential dose escalation study of cidofovir in renal and renal/pancreas transplant recipients with BKVN. The diagnosis of BKVN can be made by positive plasma PCR assay for BK virus DNA or by renal allograft biopsy demonstrating the presence of BK virus. Initiation of the study medication must take place within 60 days of the date of initial BKVN diagnosis. Based on standard of care, immunosuppressive therapy is often reduced at the time of diagnosis of BKVN. If immunosuppressive therapy was not reduced at time of BKVN diagnosis, immunosuppressive therapy will be reduced according to recently published recommendations at the time of enrollment into this trial. (See 'Guidelines for Reduction in Immunosuppression' Section 3.11 **)**. The study consists of three dose cohorts; each cohort initially will enroll approximately 12 subjects randomized 2:1 to receive either cidofovir or placebo (0.9% normal saline) in order to define the maximum tolerated dose (MTD) of these three doses. Once the MTD is established, 12 additional subjects will be enrolled at that dose. The MTD (as determined by the DSMB) is defined as the dose at which a maximum 2 of the 8 cidofovir treated subjects experience a dose limiting toxicity (DLT). Target enrollment is 48 subjects if all dose cohorts are fully enrolled. A 25% over-enrollment may be tolerated to allow for continued enrollment of subjects in a lower dose cohort while data are under review by the Data and Safety Monitoring Board (see section 3.1.3. for proposed enrollment schema) or to replace non-evaluable subjects. Study participants who have been randomized and receive cidofovir or placebo may be replaced if they discontinue the study for any reason except for toxicities, including DLTs. Patients who discontinue study medication will continue to be followed for safety through the end of the study period (day 49±3).

3.1.1 Guidelines for Reduction in Immunosuppression

It is recognized that kidney transplant recipients who develop BKVN will be receiving a range of immunosuppressive agents (and combinations) at different doses, and that the modifications (i.e. reductions) in immunosuppression after diagnosis of BKVN will vary. Although reduction in

immunosuppression is generally considered the standard of care for patients with BKVN, there is no consensus on which specific immunosuppressive agents to reduce, or by how much. In addition, there are no data to suggest that any particular strategy of immunosuppression reduction is superior to others (the goal is to reduce the "overall intensity" of the immunosuppression). However, based on clinical experience, discussions with transplant nephrologists with experience in management of BKVN, and published recommendations,[40] the following guidelines for immunosuppression reduction are provided:

- FK-506 (tacrolimus)
  - If tacrolimus is continued, reduce dose to achieve target trough level of 5-7 ng/mL
  - If tacrolimus is replaced with cyclosporine, dose cyclosoporine to achieve target trough level of 125-175 mg/mL.
- Mycophenolate mofetil
  - Reduce dose by increments of 250 mg twice daily every 2 weeks to a baseline dose of 500 mg twice daily to 0 mg/daily.

Although specific immunosuppression reduction strategies might theoretically affect the secondary efficacy analyses, the primary goals of the present study are to define safety, tolerability and MTD of cidofovir. Given the lack of consensus at the various U.S. transplant centers regarding reduction in immunosuppression, and in lieu of specific data supporting a particular strategy, we feel it is justified to allow individualization of reduction in immunosuppression for the purposes of this study. Furthermore, since the study design is blinded and placebo controlled, and a range of strategies of reduction in immunosuppression will be employed, it is expected that these strategies (which are used in day to day clinical practice) will be comparably distributed among placebo and cidofovir subjects.

3.1.2. DLT Definition.

For the purposes of this study, DLT's are defined as 1) > 50% decrease in glomerular filtration rate from baseline, 2) absolute neutrophil count (ANC) < 500 per microliter despite G-CSF. This study will be overseen by a Data and Safety Monitoring Board (DSMB) who, along with the

FDA, will review the data after each dose cohort is completed. Enrollment will not stop while the DSMB reviews the data. Data will be provided to the FDA at the same time as the DSMB to allow for concurrent review. Enrollment will proceed to the next dose cohort during the DSMB review if fewer than 3 patients develop a DLT in a cohort. As soon as a 3rd subject develops a DLT, enrollment into that cohort will stop. Subjects who are already enrolled at that dose will continue receiving study medication. Enrollment will continue at the previous dose. The DSMB will convene and determine whether continued enrollment at the dose in which a 3rd DLT occurred is acceptable. If the DSMB determines that continued enrollment at that dose is appropriate, then eligible subjects will be enrolled until the dose cohort is fully enrolled or until an unacceptably high rate of DLTs is observed. If a third study subject in cohort I experiences a DLT, study enrollment will be put on hold until the DMSB meets. If the DSMB determines that all three DLTs occurred in the cidofovir group, then further enrollment into this cohort will stop and the dose immediately prior this cohort will be considered the MTD.

3.1.3. General Plan

Only subjects who meet entry criteria will be enrolled. Potential subjects must be diagnosed as having BKVN by detection of BK virus DNA in plasma by PCR or by demonstration of BK virus inclusions in allograft renal biopsies (confirmed by immunohistochemistry, electron microscopy and/or in situ hybridization) performed as part of standard of care. BKVN histopathologic diagnosis is standard practice at most kidney transplantation centers in the United States. If a kidney biopsy is conducted, slides and samples of the biopsy tissue will be sent to the CASG Central Laboratory for subsequent distribution to and verification by a 'masked' nephropathologist at the conclusion of the study. This validation procedure will be to assess consistency of interpretation among the sites, not to confirm the initial diagnosis. Subjects diagnosed with BKVN at each institution will be consented for the study and assessed for inclusion/exclusion criteria. In addition, only those subjects with sufficient viral load in plasma (>10,000 copies/ml) to detect changes following treatment will be eligible. The plasma viral load will be determined within 21 days prior to the first dose of study drug and must be > 10,000 copies/ml. The quantitative plasma PCR to establish subject eligibility can be performed either at the local laboratory or at the CASG Virology Laboratory, per site preference. If the initial PCR to

determine subject eligibility is performed at the local site laboratory, then a pre-therapy plasma sample must also sent to the CASG Virology Laboratory and will be used for study endpoint analyses.

Additionally, plasma and urine specimen collected prior to the first doses of study drug and at subsequent follow up visits must be batched and shipped to the CASG Central Laboratory. The BK virus load in the plasma will be assessed at the CASG Virology Laboratory by Taqman PCR methodology as previously published.[16] The urine and plasma will be assessed to compare baseline and sequential post-treatment plasma and urine for changes in BK virus levels as a function of therapy.

Initially, 12 subjects will be randomized in a 2:1 ratio to receive either cidofovir or placebo (0.9% normal saline), respectively, beginning at a dose of 0.25 mg/kg (administered on days 0, 7±1, 21±1, 35±1) in Cohort I. At the completion of follow-up of Cohort I, if 0, 1, or 2 subjects experience DLTs in the cidofovir group, enrollment will continue into the next dosing Cohort II (0.5 mg/kg). Similarly, if 0, 1, or 2 subjects from the 8 cidofovir treated patients experience dose limiting toxicities in Cohort II, enrollment will continue into the next dosing Cohort III (1.0 mg/kg). If Cohort III is successfully completed with fewer than 3 subjects with cidofovir related DLTs, no further dose escalation is planned. Once the MTD has been established, an additional 12 subjects will be randomized in a 2:1 ratio to receive either cidofovir or placebo, respectively, at that dose (MTD cohort). The potential dose escalation scheme and treatment is summarized below.

Cohort	Dose	Cidofovir (n)	Placebo (n)
**I**	**0.25 mg/kg**	**8**	**4**
**II**	**0.5 mg/kg**	**8**	**4**
**III**	**1.0 mg/kg**	**8**	**4**
**IV**	**MTD (to be determined)**	**8**	**4**
		**32**	**16**

The DSMB will convene to review and analyze the data after the following prescribed times:

- After the last study participant in a dose cohort has received an entire course of study treatment
- Following a 3rd study subject experiencing a DLT in a single cohort as soon as is practically possible

FDA will be provided these data concurrently with the DSMB.

Enrolled subjects will be pre-hydrated with at least 250 mL of saline prior to receipt of study medication. Each subject will receive 4 infusions of study medication over five weeks (days 1, 7±1, 21±1, and 35±1) and followed through day 49±3 for end of study observation. All subjects will be followed closely for the development of cidofovir associated toxicities, including but not limited to renal toxicity, ocular toxicity, and neutropenia. Any pregnancies that occur while the subject is on study (through day 120±14) will be reported as serious adverse events (SAE) and followed for outcome. In addition to SAEs and cidofovir-related AEs, encoded data on pregnancy and other SAE outcomes will be reported to Gilead Sciences, Inc., the manufacturer of cidofovir.

All treated subjects will be monitored closely for changes in the GFR. Any subject who develops a >50% reduction in GFR from baseline will undergo renal biopsy as part of routine clinical care. These biopsies will be examined by an experienced nephropathologist at the local site to define etiology (rejection versus BKVN versus other condition). Any subject who develops a greater than 50% reduction in GFR will be considered to have met a DLT and will have study treatment discontinued (either cidofovir or placebo). If available, a sample of the renal biopsy and slides should be forwarded to the CASG Virology Lab for examination. A minimum of 2 unstained slides should be sent. The MOP provides detailed instructions for collecting, handling, and processing the renal biopsy and slides. At the end of the study, an independent "blinded" pathologist will evaluate the specimens.

Plasma samples will be obtained from all subjects at each dose cohort for pharmacokinetic assessment (see Section 9.5). On Day 0 and Day 35±1, blood samples will be obtained up to 20 minutes prior to the initiation of the infusion and within 15 minutes, 2 hours ±15 minutes, and 4 hours ± 15 minutes post infusion from subject in Cohorts I, II, and III. A more intensive pharmacokinetic assessment will be performed on samples from the additional 12 subjects enrolled at the MTD (MTD Cohort). Blood samples will be obtained from these subjects up to 20 minutes prior to the initiation of the infusion and at the following time points post infusion: within 15 minutes, 1 hour ± 15 minutes, 2 hours ± 15 minutes, 3 hours ± 15 minutes, and 4 hours ± 15 minutes. These samples will be obtained on infusion Day 0 and Day 35±1.

A 24-hour urine collection will be obtained from the additional 12 subjects enrolled at the MTD (MTD Cohort) to assess for excreted cidofovir levels on infusion Day 0 and Day 35±1. Urine will be collected into containers for two consecutive 12-hour periods following infusion of cidofovir. Instructions for collection, storing and shipping are located in the MOP.

A brief physical examination and laboratory evaluations will be performed at specified time points during the 5-week study period and at the 2 week follow up visit. A comprehensive physical exam will be performed on the first study visit (on days -21 to 0) and thereafter a brief physical exam will be performed at all other study visits.

3.2. Endpoints

3.2.1. Primary Endpoint

The safety and tolerability of cidofovir in kidney transplant recipients will be assessed by enumeration of adverse events reported by the subjects and/or investigator, and changes observed in the physical examination (including vital signs) and laboratory evaluations during the Drug Administration and End-of-Treatment Observation and Evaluation periods. The severity and relationship of adverse events to receipt of study drug will be determined because the primary endpoint is focusing on the safety.

3.2.2. Secondary Endpoints

- The effect of cidofovir on BK virus will be determined by:
  - percentage of subjects who achieve an undetectable BK virus urine and plasma PCR between baseline and end of treatment;
  - rate of reduction in urine and plasma BK virus load by quantitative PCR between baseline and end of treatment; and
  - time to reduction in BK virus urine and plasma PCR.
- The detailed pharmacokinetics of cidofovir will be evaluated in subjects at the maximum tolerated dose (MTD).
- The pharmacodynamics of cidofovir will be assessed by quantitating the change in BK virus DNA in urine and plasma and correlating these changes to plasma and urine levels of cidofovir between baseline and end of treatment.
- Allograft function at the completion of the study.
- Allograft rejection at the completion of the study.

3.2.3. Tertiary Endpoint

The kinetics of BK virus load in plasma and urine will be correlated with immunosuppressive medications (dose, duration and increases or decreases).

4. STUDY POPULATION

The study population is adult (≥ 18 years of age) male or female renal or renal/pancreas allograft recipients of any race with BK virus nephropathy (BKVN) diagnosed by positive plasma PCR or by renal allograft biopsy. Subjects must have no other medical condition(s) that could confound the conduct of the trial or pose a safety hazard. This study will enroll a maximum of 48 evaluable subjects at 15 to 20 study sites within the United States, Canada and/or United Kingdom. Children will not be recruited for this study because the safety database for cidofovir in children in general and specifically in children with renal transplants is insufficient to be able to assess the potential risks. In order to participate in this trial and receive study infusion, subjects must meet all inclusion and none of the exclusion criteria within 48 hours prior to infusing the initial dose of the study drug.

4.1. Inclusion Criteria

- Aged ≥ 18 years
- Kidney or kidney/pancreas transplant recipient
- New onset BKVN diagnosed by a positive plasma PCR assay for BK virus DNA or by a renal biopsy demonstrating BK virus (by immunohistochemistry, electron microscopy and/or in situ hybridization) obtained as part of standard medical care within 60 days prior to receipt of first dose of study drug.
- BK virus load in plasma > 10,000 copies/mL within prior 21 days.
- Glomerular filtration rate > 30 mL/min using Levey calculations (see Section 5.1.3.1.).
- Absolute neutrophil count >1000/µl (with GCSF support as necessary)
- Women must be post-menopausal, surgically sterile or willing to use adequate contraception (barrier method with spermicide, IUD, oral contraceptives, implant or other licensed hormone method) from time of study enrollment through 1 month after the last dose of study treatment. Men must be surgically sterile or willing to use contraception (barrier method with spermicide) from time of study enrollment through 3 months after the last dose of study treatment.

4.2. Exclusion Criteria

- Unable to obtain valid informed consent
- History of intolerance to cidofovir or related compounds (i.e. other nucleotide derivatives [adefovir or tenofovir])
- Pregnant or breast feeding women
- Prior treatment with cidofovir within the last 2 weeks
- Receipt of another investigational drug with proven nephrotoxic drug interaction with cidofovir or known antipolyoma virus activity one month prior to study entry
- Contraindication to renal biopsy (e.g., anticoagulant medication, unwilling to undergo biopsy)
- Currently receiving or anticipated to receive any of the following within 2 weeks of randomization:
  - Amphotericin preparation (intravenous)
  - Aminoglycosides (intravenous)
  - Platinum – based chemotherapeutic agents
  - NSAIDs – non steroidal anti-inflammatory drugs (aspirin given for cardioprotective treatment is acceptable up to 650 mg po daily)
  - Foscarnet
  - Pentamidine (intravenous)
  - Probenecid
  - Leflunomide
- Hypotony or uveitis

4.3. Randomization and Blinding

This is a double blind study. Subjects, investigators, and staff interacting with the subjects will be blinded to the treatment. The pharmacist will be unblinded.

Only subjects meeting entry criteria will be randomized into this study. The randomization of study subjects to the cidofovir or control group will be achieved by study-based randomization.

Randomization will be implemented by web-based randomization/enrollment system developed and maintained at the CASG Biostatistics Unit at the University of Alabama at Birmingham.

The generation and maintenance of study randomization codes will be the responsibility of the CASG Biostatisticial Unit. The codes will be kept in a secure location in the Biostatistics Unit.

Upon completion of the study, or if the study is stopped early for any reason, the study will be unblinded after all enrolled study participants have completed follow-up assessments and all data queries resolved. Unblinding may also occur when safety of a study participant is in question due to an SAE(s), or when a study participant completes the study and is under consideration for another protocol which requires knowledge of study treatment. However, under no circumstances will an investigator or other site personnel unblind a study participate without discussions with the protocol team. In non-emergent circumstances, a conference call will be held to determine whether or not un-blinding of an individual subject is necessary and appropriate. The conference call will include: CASG Principal Investigator and CASG Protocol Chair or Co-Chair, Chair of DSMB (or representative), Protocol Statistician (or representative), and NIAID DMID Project Officer (or representative). In an emergency situation in which an immediate decision is required for subject safety, a decision regarding un-blinding of an individual subject can be made by the CASG Representative or the Chair of DSMB.

4.4. Recruitment

Study participants will be identified by health care providers (nephrologists, infectious disease specialists, transplant surgeons) who are co-investigators at each site. The investigators will offer this study to their subjects who have had a kidney or kidney/pancreas transplant and have been diagnosed with BKVN. Consented study participants may receive nominal payments to offset the cost of travel, parking, and meals for study visits in accordance with local site policies and local IRB approval.

5. STUDY PROCEDURES

5.1. Clinical Evaluations

5.1.1. General Assessments

After obtaining informed consent within the 3 week period before the initial dose of study drug, a complete physical examination and medical and surgical history will be performed, including height, weight, vital signs, temperature, blood pressure, heart and respiratory rate, and an examination of all major body systems. A brief physical exam will be conducted on study visit Days 0, 7±1, 21±1, 35±1, and 49±3. The brief physical exam will consist of a review of systems, including vital signs and weight, noting those symptoms which are determined by the investigator to be clinically significant.

At each time point, assessments will be made to determine the occurrence of adverse events or serious adverse events since the previous visit. Additionally, concomitant meds and immunosuppressive meds will be reviewed and recorded at each study visit.

5.1.2. Laboratory Evaluations

A serum or urine pregnancy test will be completed immediately after obtaining consent in women of childbearing potential within 48 hours prior to receiving the initial dose of study drug (if necessary) and within 48 hours prior to each of the subsequent study drug administrations. An additional follow-up pregnancy test will be obtained on study visit (49±3). On day 120±14, a member of the study team will contact the study participant to ask if she or his partner may have become pregnant since receiving study drug.

Laboratory assessments for confirmation of BK virus include plasma and urine for PCR. The plasma and urine for PCR for BK virus will be obtained after the informed consent has been obtained (during the 3 week screening period), within 48 hours of dosing (if initial serum PCR for BK virus was obtained more than 48 hours prior to dosing), Days 7±1, 21±1, 35±1, and 49±3.

Laboratory assessments for safety must be completed at screening, within 48 hours prior to dosing for Days 0, 7±1, 21±1, 35±1, and at the last study visit day 49±3. These assessments include: a complete blood cell count (RBC count, WBC count, platelets, HCT, HgB, manual or automated WBC differential [neutrophils, basophils, monophils, eosinophils, bands if manual differential], electrolytes (sodium, potassium, chloride, bicarbonate), blood urea nitrogen (BUN), serum creatinine, total protein, glucose, albumin, total bilirubin, ALT, AST, alkaline phosphatase, calcium, magnesium, and phosphorous, ANC will be calculated at each laboratory assessment.

Urine will be collected at screening, within 48 hours prior to dosing, and at study visits on Days 7 ±1, 21±1, 35±1, and 49±3 to test for glucose, nitrite, protein, leukocyte esterase, occult blood, ketones, pH, urobilinogen, and bilirubin by dipstick. A spot urine test for a semi-quantitative urine protein/creatinine ratio will also be done at these time points.

Additional safety labs will be assessed on days 14±2, 28±2 and 42±2. These tests will include electrolytes (sodium, potassium, chloride, bicarbonate), BUN, Serum creatinine and a urine test for creatinine and protein.

Data for serum trough levels of the immunsuppressive treatment regimens for each patient as standard of care will be collected and documented in the case record form. Data to be collected will be the name of the immunosuppressive drug(s), the route, dose and frequency as well as the date and time of the last dose and the name of the assay used to measure the trough level.

5.1.3. Pharmacokinetic Studies

5.1.3.1. Clinical Pharmacology Plan

The objective of the study and overall pharmacology plan is to determine the effective cidofovir dose for treatment of BKVN in renal transplant subjects. The 3 doses are 0.25 mg/kg, 0.5 mg/kg, and 1.0 mg/kg for a total of 5 weeks (4 doses). Each subject's dose will be adjusted to reflect his/her renal function, as described in the table below. The minimum dose in each group will be either 0.25 mg/kg, 0.5 mg/kg, or 1.0 mg/kg for subjects with a CLcr of 30-60 mL/min. Within

each dose cohort, the dose is increased by one-third for participants with a CLcr between 61-90 mL/min, and increased by two thirds for subjects with a CLcr >90 mL/min. This sliding-scale dosing is designed to ensure subjects in each dose group achieve similar drug exposures (AUC) across the range of potential creatinine clearances. Procedures outlined in the protocol will be followed until the maximum tolerated dose is reached.

Most patients are expected to have CLcr between 30-60. In order to standardize exposure levels in patients with higher CLcr, adjustment of the cidofovir dose will be made according to the following table:

Cidofovir Dose Group	CLcr (mL/min)	Cidofovir Dose (mg/kg)
0.25 mg/kg	30-60	0.25
	61-90	0.33
	>90	0.42
0.5 mg/kg	30-60	0.50
	61-90	0.67
	>90	0.83
1.0 mg/kg	30-60	1.00
	61-90	1.33
	>90	1.67

Creatinine clearance may be estimated by the **Levey formula**:

170 x serum creatinine $^{-0.999}$ x age $^{-0.176}$

x 0.762 (if woman) x 1.180 (if patient is black)

x blood urea nitrogen $^{-0.170}$

x serum albumin concentration $^{0.318}$

where serum creatinine is in mg/dL, age is in years, blood urea nitrogen is in mg/dL, and serum albumin concentration is in g/dL.

To calculate GFR automatically, go to the following website:

http://www.medcalc.com/gfr.html

5.1.3.2. Cidofovir Pharmacokinetic Sampling - Limited Sample Data Collection (for dose cohorts below MTD)

5.1.3.2.1. First dose pharmacokinetics

Blood samples for determination of cidofovir in plasma will be collected following the first study dose. The cidofovir dose will be administered as a 30 minute infusion with 100 mL 0.9% (normal) saline solution. A pre-dose blood sample should be collected within 20 minutes before beginning the infusion. After the dose is administered: a post-infusion (within 15 minutes after the dose is infused) blood sample should be drawn (from the opposite arm of the infusion). Additional blood samples should be obtained at 2 hours ± 15 minutes and 4 hours ± 15 minutes post infusion.

5.1.3.2.2. Multiple post-last dose pharmacokinetics

In order to determine if undue drug accumulation is occurring, the same sample collection procedures (as in Section 5.1.3.2.1) should be performed at the time of the last dose of study drug (day 35±1).

5.1.3.2.3. Intensive Sample Data Collection for the MTD

An intensive PK assessment will be performed in the MTD Cohort. Blood samples for determination of cidofovir in plasma should be collected at time 0 (pre-dose, up to 20 minutes before); post infusion (within 15 minutes post infusion); and 1 hour ± 15 minutes, 2 hours ± 15 minutes, 3 hours ± 15 minutes, and 4 hours ± 15 minutes post infusion. This schedule of sample collection will be repeated at the time of the last dose (day 35±1) in all subjects in the MTD cohort.

5.1.3.2.4. Urine Data Collection (only for subjects at the MTD Cohort)

A 24 hour urine collection will be performed at each pharmacokinetic evaluation period. Participants are to void prior to receiving the study drug. They will be given 2 collection

containers; one for the first 12 hours following the dose and one for the second 12 hours. Participants will be instructed to void into the respective containers for 24 hours following the first dose of cidofovir. This schedule of urine collection will be performed in all subjects at the MTD cohort at 1st dose of study drug (day 1), and at last dose of study drug (day 35±1).

5.1.4. Ocular Exam

Ophthalmologic monitoring will be performed by an Eye Care Professional. Ocular pressure and slit-lamp examination will be performed at baseline (within 3 weeks prior to study drug infusion), and within 2 weeks after the last dose. Ophthalmologic examination will be performed for any reported ocular symptoms at any time during the study.

5.1.5. Renal Biopsy

Renal biopsy will be performed during the study period in any subject who develops a greater than 50% reduction in glomerular filtration rate from the initial pre-treatment GFR (48 hours prior to first study drug administration), unless that patient has had a renal biopsy as part of standard of care within the previous 7 days.

For patients who have had an allograft kidney biopsy for standard of care during the study period, a repeat biopsy will be performed only if the both of the following circumstances occur:

- at least 7 days have passed since the standard of care biopsy was conducted, and
- the GFR decreases ≥50% from baseline and has decreased at least 15% from the GFR at the time of the standard of care biopsy.

If renal biopsies are obtained as part of the standard of care for the patient, these also will be reported and remnant renal tissue specimen obtained, if possible. Any remaining specimen and slides from the protocol-indicated or clinically-indicated renal biopsies should be sent to the CASG Central Laboratory. Guidelines on the collection and sample handling for renal biopsies are contained within the MOP for this study.

5.1.6. Assessment of Emergence of Resistance to Cidofovir

A urine sample for cytomegalovirus (CMV) culture will be obtained at baseline before administration of study treatment. An aliquot of the urine sample will be shipped to the CASG Virology Lab for processing at baseline and at Day 49 ± 3. If the urine sample is positive for CMV culture at baseline in the CASG virology lab, then the day 49 ± 3 sample will be tested. After the study is completed, all of the positive CMV isolates will be analyzed for genotypic and/or phenotypic evidence of cidofovir resistance. Instructions for handling the specimen will be described in the MOP.

Samples obtained for BK virus assessments may be retained to determine the development of cidofovir resistant BK virus as the technology develops.

5.1.7. Pregnancy Prevention Counseling

The package insert recommends that women continue to use effective birth control for at least one month and that men continue to use adequate birth control for 3 months following cidofovir use. At screening and visit days 0 (within 48 hours of dosing), 7±1(within 48 hours of dosing), 21±1(within 48 hours of dosing), 35±1 and 49±3, the PI or his/her designee will review the requirement for use of adequate birth control as stated in the inclusion and exclusion criteria and in the signed informed consent form. Additionally, the study participant will be provided with an information sheet reiterating the required adequate birth control method for non-surgically sterilized and non-post menopausal study participants. In addition to the end of study visit pregnancy test (day 49±3), a phone call will be made to all patients (male and female) on day 120±14 to ask about any pregnancies. Known pregnancies in patients will be followed for outcome. Men will be given contact information for their partners if the partner wishes to disclose information about the pregnancy and its outcome.

5.1.8. Specimen Preparation, Handling and Shipping

5.1.8.1. Instructions For Specimen Preparation, Handling and Storage.

Guidance for specimen preparation, handling and storage will be found in the MOP for all specimens other than safety laboratory studies and pregnancy tests. Safety laboratory tests and pregnancy tests will be managed per site specific institutional requirements.

5.1.8.2. Specimen Shipment

Instructions are in the MOP for shipment of all required specimens other than safety lab and pregnancy tests to the central laboratory for further processing or distribution.

5.2. Schedule of Procedures (see Appendix A)

Patients with BKVN diagnosed by positive plasma PCR or renal allograft biopsy are eligible for the study. Study participants who provide written informed consent will be screened to determine final eligibility for participation in this study. If available, samples of renal biopsy (samples and biopsy material) from which the diagnosis of BKVN is made will be sent to the CASG Central Laboratory for re-evaluation by a "blinded" histopathologist to evaluate potential inter-center variations in interpretation. After subjects are consented, the following screening procedures will be performed during the period starting 2 weeks prior to the first study treatment through Day 49±3 after first dose of study drug.

5.2.1. Day: (–) 21 to 0

Obtain informed consent from subject or subject's legally authorized representative.

5.2.1.1. Screening period: (-) 21 to Day 0

- Verify that Inclusion and Exclusion criteria have been met
- Pregnancy test
- Counsel regarding avoidance of pregnancy
- Verify positive plasma PCR or renal allograft biopsy diagnosis of BKVN (initial diagnosis within 60 days prior to first study infusion)
- Obtain Medical and Surgical History
- Complete Comprehensive Physical Examination and Vital Signs (BP, HR, RR and T)
- Obtain urine for
  - BK viral load
  - Dipstick test for:
    - pH
    - Protein
    - Glucose
    - Ketones
    - Bilirubin
    - Blood
    - Nitrite
    - Leukocyte Esterase

- Urobilinogen
- Spot urine for:
  - Quantitative urine protein/creatinine ratio
- Obtain Baseline Safety Labs:
  - Complete blood cell count
    - Platelet count
    - Hematocrit
    - Hemoglobin
    - RBC
    - WBC count with differential (manual or automated)
      - Segs/Neurtophils
      - Bands
      - Lymphocytes
      - Monocytes
      - Eosinophils
      - Basophils
  - Blood urea nitrogen (BUN)
  - Serum creatinine
  - Electrolytes
    - Sodium
    - Potassium
    - Chloride
    - Bicarbonate
  - Calcium
  - Magnesium
  - Phosphorous
  - Total protein
  - Glucose
  - Albumin
  - Total bilirubin
  - ALT
  - AST
  - Alkaline phosphatase
- Calculate ANC = (neutrophils + bands) x (WBC X 10)
- Calculate GFR
- Obtain Plasma for BK Viral Load
- General Assessments:
  - Document concomitant medications
  - Document concomitant immunosuppressive therapy (including changes in doses)
- Ocular exam (within 3 weeks prior to first study drug infusion)
  - Measure the ocular pressure
  - Slit lamp examination

5.2.1.2. Within 48 hours prior to dosing unless otherwise stated. (If any assessments required in section 5.2.1.1 have been done within 48 hours of dosing, the duplicative assessments below should not be repeated [e.g. Inclusion and Exclusion Criteria, pregnancy test, brief physical exam, urinalysis, etc.])

- Review Inclusion and Exclusion criteria
- Verify GFR is >30mL/min using Levey calculations (see Section 5.1.3.1)
- Verify that ANC > 1000 µl (with GCSF support as necessary)
- Counsel regarding avoidance of pregnancy
- Obtain study participant's weight in kilograms
- Randomize patient to active treatment or placebo
- Obtain serum or urine for pregnancy test within 48 hours prior to study drug infusion– if positive, patient is excluded from study
- Brief Physical Examination and Vital Signs (BP, HR, RR and T)
- Obtain urine for:
  - BK viral load
  - Dipstick test for:
    - pH
    - Protein
    - Glucose
    - Ketones
    - Bilirubin
    - Blood
    - Nitrite
    - Leukocyte Esterase
    - Urobilinogen
  - Spot urine for:
    - Future testing for CMV culture
    - Quantitative urine protein/creatinine ratio
- Obtain Baseline Safety Labs:
  - Complete blood cell count
    - Platelet count
    - Hematocrit
    - Hemoglobin
    - RBC
    - WBC count with differential (manual or automated)
      - Segs/Neutrophils
      - Bands
      - Lymphocytes
      - Monocytes
      - Eosinophils
      - Basophils
  - Platelet count

- Blood urea nitrogen (BUN)
- Serum creatinine
- Electrolytes
  - Sodium
  - Potassium
  - Chloride
  - Bicarbonate
- Calcium
- Magnesium
- Phosphorous
- Total protein
- Glucose
- Albumin
- Total bilirubin
- ALT
- AST
- Alkaline phosphatase

- Calculate ANC = (neutrophils + bands) x (WBC X 10)
- Calculate GFR
- Obtain Plasma for BK Viral Load
- Obtain Blood for laboratory test
  - Record immunosuppressive drug trough levels if obtained for clinical standard of care
- General Assessments:
  - Record concomitant medications
  - Record concomitant immunosuppressive therapy (including changes in dosages)

5.2.2. Day 0 (dosing)

- PK 24 hour urine for MTD Cohort only
  - Void before beginning study drug infusion
  - Collect urine for 24 hours after start of infusion in 2 twelve hour containers
- Pre-hydrate with at least 250 mL of saline (generally over 15-30 minutes)
- Infuse study treatment (over approximately 30 minutes)
- Obtain plasma specimens for PK assessments
  - For Cohorts I, II, and III
    - Obtain blood for plasma
      - at baseline (within 20 minutes prior to beginning infusion)
      - within 15 minutes post infusion
      - 2 hours ± 15 minutes post infusion
      - 4 hours ± 15 minutes post infusion
  - In MTD Cohort
    - Obtain blood for plasma
      - at baseline (within 20 minutes prior to beginning infusion)

    - within 15 minutes post infusion
    - at 1 hour ± 15 post infusion
    - 2 hours ± 15 minutes post infusion
    - 3 hours ± 15 minutes post infusion
    - 4 hours ± 15 minutes post infusion
- General Assessments:
  - Record adverse events
  - Record concomitant medications
  - Record concomitant immunosuppressive therapy

5.2.3. Day 7 ± 1 (unless otherwise indicated)

- Brief Physical Examination and Vital Signs (Weight, BP, HR, RR and T)
- Counsel regarding avoidance of pregnancy
- Obtain study participant's weight in kilograms
- Obtain urine for
  - BK viral load
  - Dipstick test for:
    - pH
    - Protein
    - Glucose
    - Ketones
    - Bilirubin
    - Blood
    - Nitrite
    - Leukocyte Esterase
    - Urobilinogen
  - Spot urine for:
    - Quantitative urine protein/creatinine ratio
- Obtain Baseline Safety Labs:
  - Complete blood cell count
    - Platelet count
    - Hematocrit
    - Hemoglobin
    - RBC
    - WBC count with differential (manual or automated)
      - Segs/Neutrophils
      - Bands
      - Lymphocytes
      - Monocytes
      - Eosinophils
      - Basophils
  - Platelet count

  - Blood urea nitrogen (BUN)
  - Serum creatinine
  - Electrolytes
    - Sodium
    - Potassium
    - Chloride
    - Bicarbonate
  - Calcium
  - Magnesium
  - Phosphorous
  - Total protein
  - Glucose
  - Albumin
  - Total bilirubin
  - ALT
  - AST
  - Alkaline phosphatase
- Calculate ANC = (neutrophils + bands) x (WBC X 10)
- Calculate GRF (if DLT level is reached, do not infuse study drug)
- Obtain serum or urine for pregnancy test within 48 hours prior to study drug infusion– if positive, patient is excluded from study
- Obtain Blood for laboratory test
  - Plasma for BK viral load
  - Record immunosuppressive drug trough levels if obtained for clinical standard of care
- Pre-hydrate with at least 250 mL of saline (approximately 15-30 minutes)
- Infuse study treatment (approximately 30 minutes)
- Tests contingent on symptoms
  - Renal Biopsy (obtain results if done for clinical reasons or schedule if a reduction in GFR > 50% from baseline)
  - Ocular exam
- General Assessments:
  - Record adverse events
  - Record concomitant medications
  - Record concomitant immunosuppressive therapy (including changes in dosage levels)

5.2.4. Day 14 ± 2 (blood may be obtained at remote labs and shipped to local site)

- Obtain Serum Safety Labs
  - Electrolytes (sodium, potassium, chloride, bicarbonate)
  - Blood urea nitrogen
  - Serum creatinine
- Obtain Urine Safety Labs
  - Creatinine

  - Protein

5.2.5. Day 21 ± 1 (unless otherwise indicated)

- Brief Physical Examination and Vital Signs (Weight, BP, HR, RR and T)
- Counsel regarding avoidance of pregnancy
- Obtain study participant's weight in kilograms
- Obtain urine for
  - BK viral load
  - Dipstick test for:
    - pH
    - Protein
    - Glucose
    - Ketones
    - Bilirubin
    - Blood
    - Nitrite
    - Leukocyte Esterase
    - Urobilinogen
  - Spot urine for:
    - Quantitative urine protein/creatinine ratio
- Obtain Baseline Safety Labs:
  - Complete blood cell count
    - Platelet count
    - Hematocrit
    - Hemoglobin
    - RBC
    - WBC count with differential (manual or automated)
      - Segs/Neutrophils
      - Bands
      - Lymphocytes
      - Monocytes
      - Eosinophils
      - Basophils
  - Platelet count
  - Blood urea nitrogen (BUN)
  - Serum creatinine
  - Electrolytes
    - Sodium
    - Potassium
    - Chloride
    - Bicarbonate
  - Calcium

- Magnesium
- Phosphorous
- Total protein
- Glucose
- Albumin
- Total bilirubin
- ALT
- AST
- Alkaline phosphatase

- Calculate ANC = (neutrophils + bands) x (WBC X 10)
- Calculate GFR (if DLT level is reached, do not infuse study drug)
- Obtain serum or urine for pregnancy test within 48 hours prior to study drug infusion– if positive, patient is excluded from study
- Obtain Plasma for BK Viral Load
- Obtain Blood for laboratory tests
  - Record immunosuppressive drug trough levels if obtained for clinical standard of care
- Pre-hydrate with at least 250 mL of saline (approximately 15-30 minutes)
- Infuse study treatment (approximately 30 minute infusion)
- Tests contingent on symptoms
  - Renal Biopsy (obtain results if conducted for clinical reasons or schedule if a reduction in GFR > 50% from baseline is observed)
  - Ocular exam
- General Assessments:
  - Record adverse events
  - Record concomitant medications
  - Concomitant immunosuppressive therapy

5.2.6. Day 28 ± 2 (blood samples may be obtained at remote labs and shipped to the site lab)

- Obtain Serum Safety Labs
  - Electrolytes (sodium, potassium, chloride, bicarbonate)
  - Blood urea nitrogen
  - Serum creatinine
- Obtain Urine Safety Labs
  - Creatinine
  - Protein

5.2.7. Day 35 ± 1 (unless otherwise indicated)

- Brief Physical Examination and Vital Signs (Weight, BP, HR, RR and T)
- Counsel regarding avoidance of pregnancy

- Obtain study participant's weight in kilograms
- Obtain urine for
  - BK viral load
  - Dipstick test for:
    - pH
    - Albumin
    - Protein
    - Glucose
    - Ketones
    - Bilirubin
    - Blood
    - Nitrite
    - Leukocyte Esterase
    - Urobilinogen
  - Spot urine for:
    - Quantitative urine protein/creatinine ratio
- Obtain Baseline Safety Labs:
  - Complete blood cell count
    - Platelet count
    - Hematocrit
    - Hemoglobin
    - RBC
    - WBC count with differential (manual or automated)
      - Segs/Neutrophils
      - Bands
      - Lymphocytes
      - Monocytes
      - Eosinophils
      - Basophils
  - Platelet count
  - Blood urea nitrogen (BUN)
  - Serum creatinine
  - Electrolytes
    - Sodium
    - Potassium
    - Chloride
    - Bicarbonate
  - Calcium
  - Magnesium
  - Phosphorous
  - Total protein
  - Glucose
  - Albumin
  - Total bilirubin
  - ALT

  - AST
  - Alkaline phosphatase
- Calculate ANC = (neutrophils + bands) x (WBC X 10)
- Calculate GFR (if DLT level is reached, do not infuse study drug)
- Obtain serum or urine for pregnancy test within 48 hours prior to study drug infusion– if positive, patient is excluded from study
- Obtain Plasma for BK Viral load
  - Record immunosuppressive drug trough levels if obtained for clinical standard of care
- PK 24 hour urine for MTD cohort
  - Void before beginning study drug infusion
  - Collect urine for 24 hours after start of infusion
- Pre-hydrate with at least 250 mL of saline
- Infuse study treatment (30 minute infusion)
- Obtain plasma specimens for PK assessments
  - For Cohorts I, II, and III
    - Obtain blood for plasma
      - at baseline (within 20 minutes prior to beginning infusion)
      - within 15 minutes post infusion
      - 2 hours ± 15 minutes post infusion
      - 4 hours ± 15 minutes post infusion
  - For the MTD Cohort
    - Obtain blood for plasma
      - at baseline (within 20 minutes prior to beginning infusion)
      - within 15 minutes post infusion
      - 1 hour ± 15 post infusion
      - 2 hours ± 15 minutes post infusion
      - 3 hours ± 15 minutes post infusion
      - 4 hours ± 15 minutes post infusion
- Tests contingent on symptoms
  - Renal Biopsy (obtain results if biopsy is done for clinical reasons or schedule if a reduction in GFR > 50% from baseline is observed)
  - Ocular exam by Eye Care Professional (within 2 weeks after Day 35±1)
    - measure the ocular pressure
    - slit lamp examination
- General Assessments:
  - Record adverse events
  - Record concomitant medications
  - Record concomitant immunosuppressive therapy (including dosage levels)

5.2.8. Day 42 ± 2 (blood samples may be obtained at remote labs and shipped to the site lab)

- Obtain Serum Safety Labs

  - Electrolytes (sodium, potassium, chloride, bicarbonate)
  - Blood urea nitrogen
  - Serum creatinine
- Obtain Urine Safety Labs
  - Creatinine
  - Protein

5.2.9. Day 49 ± 3

- Brief Physical Examination and Vital Signs (Weight, BP, HR, RR and T)
- Counsel regarding avoidance of pregnancy
- Obtain serum or urine for pregnancy test
- Obtain urine for:
  - BK viral load
  - Dipstick test for:
    - pH
    - Protein
    - Glucose
    - Ketones
    - Bilirubin
    - Blood
    - Nitrite
    - Leukocyte Esterase
    - Urobilinogen
  - Spot urine for:
    - Quantitative urine protein/creatinine ratio
- Future testing for CMV
- Obtain Baseline Safety Labs:
  - Complete blood cell count
    - Platelet count
    - Hematocrit
    - Hemoglobin
    - RBC
    - WBC count with differential (manual or automated)
      - Segs/Neutrophils
      - Bands
      - Lymphocytes
      - Monocytes
      - Eosinophils
      - Basophils
  - Platelet count
  - Blood urea nitrogen (BUN)
  - Serum creatinine

- Electrolytes
  - Sodium
  - Potassium
  - Chloride
  - Bicarbonate
- Calcium
- Magnesium
- Phosphorous
- Total protein
- Glucose
- Albumin
- Total bilirubin
- ALT
- AST
- Alkaline phosphatase

- Calculate ANC = (neutrophils + bands) x (WBC X 10)
- Calculate GFR
- Obtain Plasma for BK Viral Load
  - Record immunosuppressive drug trough levels if obtained for clinical standard of care
- Tests contingent on symptoms
  - Renal Biopsy (obtain results if biopsy done for clinical reasons or schedule if a reduction in GFR > 50% from baseline is observed)
- General Assessments:
  - Record adverse events
  - Record concomitant medications
  - Record concomitant immunosuppressive therapy

5.2.10. Day 120 ± 14

Phone call from study coordinator inquiring about pregnancy status. Female study participants to be followed to outcome. Men will be given contact information for their partners if the partner wishes to disclose information about the pregnancy and its outcome.

5.3. Early Termination During Weeks 1-5

If a subject withdraws or is discontinued from the study at any time prior to Week 5 (See Section 6.1) and has received any study medication, the subject is requested to return for all clinical assessments including collection of blood and urine samples for BK virus PCR. SAEs and AEs will be followed according to guidelines in Section 8.1 of this protocol.

6. SUBJECT & STUDY DISCONTINUATION

6.1. Subject Discontinuation

Every effort consistent with subject safety and choice will be made to ensure that each subject completes the study. However, subjects may be discontinued from the study medication for the following reasons:

- Subject develops a decrease in neutrophil count of < 500 cells /µL that is suspected to be related to the study drug
- Subject develops renal impairment with > 50% reduction in GFR compared to baseline that is suspected to be related to study drug
- Subject develops ocular symptoms (such as hypotony [>50% reduction in intraocular pressure] or uveitis) that are suspected to be related to study drug
- Subject does not comply with the protocol
- There is an unacceptable adverse event (Refer to NCI CTC [National Cancer Institute Common Toxicity Criteria – also referred to as the CTCAE] toxicity scale found in the MOP)
- Investigator recommends discontinuation and documents the reason(s)
- Subject’s decision to discontinue for any reason
- There is a need for a medical intervention that is not allowed by the protocol
- The CASG, NIH and Gilead Sciences Inc.’s decision with documented reason(s) following discussion with the principal investigator
- Pregnancy

6.2. Study Discontinuation

The CASG, NIH, DSMB and Gilead Sciences, Inc. have the right to terminate this study at any time. Reasons for terminating the study may include, but are not limited to, the following:

- The incidence or severity of adverse events in this or other studies indicates a potential health hazard to subjects
- Subject enrollment is unsatisfactory
- Data recording is inaccurate or incomplete
- Investigator does not adhere to the protocol or applicable regulatory guidelines in conducting the study

7. STUDY TREATMENT

7.1 Study Products

7.1.1 Cidofovir (VISTIDE®)

Cidofovir (VISTIDE®) is a sterile, hypertonic aqueous solution for intravenous infusion only. The solution is clear and colorless. It is supplied in clear glass vials, each containing 375 mg of anhydrous cidofovir (VISTIDE®) in 5.0 mL aqueous solutions at a concentration of 75 mg/mL. The formulation is pH-adjusted to 7.4 with sodium hydroxide and/or hydrochloric acid and contains no preservatives. The appropriate volume of cidofovir (VISTIDE®) must be removed from the single-use vial and diluted within 24 hours of administration (see product administration section 7.6).

7.1.2. Placebo

Normal (0.9%) sterile saline for intravenous use is the placebo for this study and will be obtained at each site from the site's pharmacy.

7.2. Packaging and Labeling

Cidofovir (VISTIDE®) will be supplied in its commercial packaging.

7.3. Storage and Handling

7.3.1. Cidofovir (VISTIDE®)

Cidofovir (VISTIDE®) should be stored at controlled room temperature 20-25° C (68- 77° F). Based on USP definitions, the controlled room temperature where the cidofovir is stored may vary briefly between 15° C and 30° C (59° – 86° F). Due to mutagenic properties of cidofovir (VISTIDE®), adequate precautions including the use of appropriate safety equipment are recommended for the preparation, administration, and disposal of cidofovir (VISTIDE®). The National Institutes of Health presently recommends that such agents be prepared in a Class II laminar flow biological safety cabinet and that personnel preparing drugs of this class wear

surgical gloves and a closed front surgical-type gown with knit cuffs. If cidofovir (VISTIDE®) contacts the skin, wash membranes and flush thoroughly with water.

7.3.2. 0.9% Normal Saline

0.9% normal saline should be stored in accordance with local pharmacy requirements.

7.4. Disposal

Excess or residual cidofovir (VISTIDE®) and all other material used in the admixture preparation and administration should be placed in a leak-proof, puncture-proof container and disposed of per institutional standards. The disposal should be documented on the drug accountability form.

7.5. Drug Accountability

After receipt of the pre-packaged drugs and supplies, the site pharmacist has responsibility for distribution of the products to the Principal Investigator or his/her designee, and has ultimate responsibility for drug accountability. Each pharmacist will be responsible for and maintain logs of receipt, storage conditions and disposal of study drug. These documents will be maintained in a secure and accessible location. At study completion, the sites will be required to return the drug accountability log to the CASG Central Unit upon request, retaining a copy of the drug accountability form in their study files.

7.6. Study Drug Preparation and Administration

The amount of cidofovir (VISTIDE®) will be calculated by the pharmacist based on the dose cohort and study participant's weight in kilograms. Dose adjustment for renal insufficiency is permissible according to the formula in section 7.7.

Cidofovir (VISTIDE®) Preparation (Preparation to be done by the site pharmacists who is not blinded to treatment)

- Inspect the vial visually for particulate matter and discoloration prior to administration. If particulate matter or discoloration is observed, DO NOT use the vial.

- Calculate the dose needed for the given cohort based on the subject's weight and CLcr if necessary (see section 7.7).
- With a syringe, extract the appropriate volume of cidofovir (VISTIDE®) from the vial and transfer the dose to an infusion bag containing 100 mL 0.9% normal saline solution. (Please note, compatibility of cidofovir with Ringer's solution, Lactated Ringers solution or bacteriostatic infusion fluids has not been evaluated and should not be used for the infusion).

Cidofovir (VISTIDE®) infusion admixtures must be administered within 24 hours of preparation. If the infusion admixture is not administered immediately, store for no more than 24 hours under refrigeration (2-8°C). Allow any refrigerated infusion admixture to equilibrate to room temperature prior to administration.

DO NOT add other drugs or supplements to the cidofovir (VISTIDE®) admixture for concurrent administration.

Administration of cidofovir (VISTIDE®) or placebo

- Subject should be hydrated with at least 250 mL of 0.9% normal saline infused over a 15-30 minute period immediately prior to administration of cidofovir (VISTIDE®) or placebo. If volume depleted, subject's fluid status should be corrected and reassessed until adequate urine output is assessed.
- Cidofovir (VISTIDE®) prepared as outlined above must be infused at a constant rate over a period of approximately 30 minutes. Use of a standard infusion pump or another constant rate infusion device is recommended.

7.7. Dose Modification

Dose modifications are only permitted for specified adjustments for renal function.

Most patients are expected to have CLcr between 30-60. In order to standardize exposure levels in patients with higher CLcr, adjustment of the cidofovir dose will be made according to the following table:

Cidofovir Dose Group	CLcr (mL/min)	Cidofovir Dose (mg/kg)
0.25 mg/kg	30-60	0.25
	61-90	0.33
	>90	0.42
0.5 mg/kg	30-60	0.50
	61-90	0.67
	>90	0.83
1.0 mg/kg	30-60	1.00
	61-90	1.33
	>90	1.67

7.8. Withdrawal of Study Drug

Subjects who cannot tolerate the study medication will be discontinued from additional treatment.

7.9. Concomitant Therapy

All medications required for the care of each subject during this study are permitted except for those listed in Section 7.10 should be noted on the appropriate Case Record Forms(s) (CRF). All concomitant medications MUST be recorded on the appropriate CRF pages. The generic name of all medications, the dosage, the route of administration, the frequency, the duration of administration and the indication will be recorded in the appropriate sections of the CRF. Each time an indication changes, a new entry must be made on the appropriate page of the CRF.

7.10. Prohibited Therapies

The following medications are not permitted for subjects from enrollment through Day 49:

- Any amphotericin preparation (intravenous)
- An aminoglycoside (intravenous)
- Platinum – based chemotherapeutic agents
- NSAIDS non steroidal anti-inflammatory drugs (aspirin given for cardioprotective treatment is acceptable up to 650 mg po daily)
- Foscarnet

- Pentamidine (intravenous)
- Probencid
- Leflunomide

8.0. ASSESSMENT OF SAFETY

The investigator is responsible for reporting all adverse events (AE) and serious adverse events (SAE) that are observed or reported during the study, regardless of relationship to study product. At each clinical and safety evaluation during the treatment and follow-up period (through day 49± 3), the investigator or site personnel should document any AEs or SAEs, as detailed in this protocol. The investigator should include his/her assessment of any SAEs resulting from study participation (e.g., complications resulting from the procedures or from treatment). Specific instructions on where to record AEs are provided in the MOP for this study. The study coordinator or other research staff as designated by the PI may complete and document the AEs. Specific definition and reporting information for AEs and SAEs is summarized below.

8.1. Adverse event

ICH E6 Good Clinical Practice Guidelines defines an AE as any untoward medical occurrence in a patient or clinical investigation subject administered a pharmaceutical product regardless of its causal relationship to the study treatment. An AE can therefore be any unfavorable and unintended sign (including a clinically significant abnormal laboratory finding), symptom, or disease temporally associated with the use of medicinal (investigational) product. The occurrence of an adverse event may come to the attention of study personnel during study visits and interviews or by a study recipient presenting for medical care.

All AEs must be graded for intensity using the National Cancer Institute Toxicity criteria (located in the MOP) and assigned relationship to study product. All AEs will be followed until satisfactory resolution or until the Principal Investigator or Sub-investigator deems the event to be chronic or the patient to be stable.

Clinical adverse events are illnesses, signs, or symptoms that have appeared or worsened during the acute phase of the study or during the follow-up period. These may include the following: 1) the exacerbation of a pre-existing illness, 2) an increase in the frequency or intensity of a pre-existing episodic event or condition, 3) any condition detected or diagnosed after study drug administration even though it may have been present prior to the start of the study or 4)

continuous persistent disease or symptoms present at baseline that worsen following the start of the study.

Any medical condition or clinically significant laboratory abnormality with an onset date prior to the infusion of the initial dose of study drug is considered to be pre-existing and should be documented on the Medical History source document. Any AE (i.e., a new event or an exacerbation of a pre-existing condition) from the time the initial study drug is infused and onward should be recorded on the Adverse Events CRF, as specified in the MOP. AEs, whether believed to be treatment-related or not, must be documented on the Adverse Event page of the case report forms as outlined in the MOP. Any releveant concomitant medicines to the event should be documented. Relationship to study products and severity of AEs, regardless of cause, should be graded by the investigator as outlined below:

Relationship to study products: The investigator's assessment of an AE's relationship to study drug is part of the documentation process, but it is not a factor in determining what is or is not reported in the study. If there is any doubt as to whether a clinical observation is an AE, the event should be reported. All adverse events must have their relationship to study product assessed using the following terms: associated or not associated. In a clinical trial the study product must always be suspect. To help assess, the following guidelines are used.

- Associated – There is a known temporal relationship *and/or*, if re-challenge is done, the event abates with de-challenge and reappears with re-challenge *and/or* the event is known to occur in association study product or with a product in a similar class of study products

- Not Associated -the AE is completely independent of study product administration; and/or evidence exists that the event is definitely related to another etiology.

Intensity of Event: All adverse events will be assessed by the investigator using the NCI CTC Toxicity Scale (see MOP). For events not included in the protocol defined grading system, then the following guidelines will be used to quantify intensity.

- Mild: events require minimal or no treatment and do not interfere with the patient's daily activities.
- Moderate: events result in a low level of inconvenience or concern with the therapeutic measures. Moderate events may cause some interference with functioning.
- Severe: events interrupt a patient's usual daily activity and may require systemic drug therapy or other treatment. Severe events are usually incapacitating.
- Life threatening: Any adverse drug experience that places the patient or subject, in the view of the investigator, at immediate risk of death from the reaction as it occurred, i.e., it does not include a reaction that had it occurred in a more severe form, might have caused death.

Changes in the severity of an AE should be documented to allow an assessment of the duration of the event at each level of intensity to be performed. Adverse events characterized as intermittent require documentation of onset and duration of each episode.

8.2. Serious Adverse Event

An SAE is defined as an AE meeting one of the following conditions:

- Death during the period of protocol defined surveillance
- Life Threatening Event (defined as a subject at immediate risk of death at the time of the event)
- An event requiring inpatient hospitalization or prolongation of existing hospitalization during the period of protocol defined surveillance
- Results in congenital anomaly or birth defect
- Results in a persistent or significant disability/incapacity
- Any other important medical event that may not result in death, be life threatening, or require hospitalization, may be considered a serious adverse experience when, based upon

appropriate medical judgment, the event may jeopardize the subject and may require medical or surgical intervention to prevent one of the outcomes listed above. Examples of such medical events include allergic bronchospasm requiring intensive treatment in an emergency room or at home, blood dyscrasias or convulsions that do not result in inpatient hospitalization, or the development of drug dependency or drug abuse.

8.3. Reporting Requirements

FDA regulations (21 CFR 312.64 and 312.66) require that an investigator notify the sponsor and the local IRB promptly of any serious adverse event, deaths, or life-threatening problems that may reasonably be regarded as caused by or associated with the administration of the investigational drug. Adverse events will be reported to the FDA in compliance with 21 CFR 312.32 and entered into the internet data system. Except for pregnancy, assessing AEs and SAEs will occur through day 49±3 visit. Pregnancy will be assessed through day 120±14.

8.3.1. Reporting Adverse Events

Adverse Events including local and systemic reactions not meeting the criteria for "serious adverse events" should be captured on the appropriate case report form (CRF). Information to be collected includes event description, time of onset, investigator assessment of severity, relationship to study product, time of resolution of the event, seriousness, and outcome. All AEs occurring while on study must be documented appropriately regardless of relationship. All AEs will be followed to adequate resolution.

Any medical condition that is present at the time that the patient is screened should be considered as pre-existing and should not be reported as an AE. However, if there is a change in the intensity (worsening of symptoms) at any time during the study it should be recorded as an AE.

The investigator should document the occurrence of an AE on the AE CRF. AE CRFs should be submitted to the CASG Central Unit monthly or when requested. The CASG, FDA, the

DMID/NIAID/NIH, Gilead Sciences, Inc., the IRB, or the DSMB may request additional reports on adverse experiences at any time.

8.3.2. Reporting Serious Adverse Events

Any AE considered serious by the Principal Investigator or Co-investigator or which meets the aforementioned criteria (see Section 8.2.) must be submitted on the SAE form provided by the CASG Central Unit.

For those events meeting the previously described definition of SAE, the completion of an SAE form is required. Specific information regarding where to send this form is included in the MOP for this study. All serious adverse events will be:

- recorded on the appropriate Serious Adverse Event case report form
- followed through resolution by a study physician
- reviewed and credentialed by a study physician prior to submission of the form

Life-threatening SAEs or death must be reported to the CASG Central Unit within **one business day** of site awareness of the event by calling 877-975-7280 and faxing (205-934-8559) a completed "Serious Adverse Event Report", including a description of the event and the investigator's judgment of the causality, to the CASG Central Unit Clinical Administrator. This should be sent even when the full data relating to the case have not yet been collected. The Central Unit will then forward this report to DMID within 24 hours of receipt of the report.

All other SAEs must be reported via telephone to the CASG Central Unit Clinical Administrator within **2 business days** of site awareness of the event. All SAEs will be reported as required to all sites participating Institutional Review Board (IRB). A copy and paper trail of the report to the IRB must be maintained in the Project Notebook.

Within **7 business days if possible**, a full written description of the serious adverse event and any sequelae should be submitted to the CASG Central Unit. The CASG Central Unit is responsible for submitting the SAE and any follow up information to the DMID Project Officer, the DMID

Medical Monitor or designated contact. The report is then forwarded to the DMID, Gilead Sciences, Inc., Office of Clinical Affairs for review and the Office of Regulatory Affairs for submission to FDA.

The investigator should exercise medical and scientific judgment when deciding whether expedited reporting is appropriate in other situations not strictly meeting the SAEs criteria described above. Important medical events that may meet SAE criteria include intensive treatment in an emergency room, convulsions that do not result in hospitalizations, or any clinical or laboratory event deemed serious by the site investigator.

If new or follow up information is received concerning a participant's SAE, the site should complete a follow up SAE report, signed and dated by the investigator. The investigator is responsible for continuing to report new or relevant information related to the SAE. Follow up information should be sent to the Central Unit as outlined in the MOP for this study.

The investigator and supporting personnel responsible for the study participant's care should institute any supplemental investigations of SAEs based on their clinical judgment of likely causative factors. This may include extra clinical laboratory tests, histopathological examination, or consulting an appropriate specialist. DMID or the CASG Central Unit may also request the investigator conduct supplemental assessments. The results of any additional assessments conducted must be reported to the CASG Central Unit. Any study participants with a SAE will be followed clinically, and by laboratory parameters if appropriate, until all parameters return to baseline or are considered irreversible or 30 days after the last dose of study medication and the patient has completed all study visits. If a study participant dies during participation in the study, an autopsy should be requested and a copy of the autopsy reports must be submitted to the CASG Central Unit. If an autopsy is denied, it should be noted in the participant's study files.

The investigator is responsible for promptly notifying his/her IRB of all SAEs as per their IRB SOPs. This includes follow up information, occurring at his/her site and any SAE regulatory report, including any follow up reports that he/she receives from DMID or the CASG Central Unit. SAE's are required to be reported under expedited procedures.

8.3.3. Flowchart Summarizing AE and SAE Reporting

Schedule of Serious Adverse Event Reporting and Adverse Event Reporting:

For SAEs and AEs that occur:

If death or life threatening event

The site will:

Contact the CASG Central Unit by phone or email within 1 business day of knowledge of event
Complete 3 page SAE form
Fax form to CASG Central Unit within 1 business day of knowledge of event
Report SAE to site IRB (per local IRB guidelines)
Log SAE on Adverse Event CRF
Submit original report within 5 business days to the CASG CU
If warranted, follow up to the initial SAE report should be completed and submitted to the CASG CU in 7 business days of initial report.

The CASG central unit will:

Submit the SAE report to DMID Project Officer, PPD, Gilead Sciences, Inc. and the CASG Biostatistics Unit within 1 business day of receipt of fax.

If another SAE, as described in above list (section 8.2.)

The site will:

Contact the CASG Central Unit by phone or email within 2 business days of knowledge of event
Complete 3 page SAE form
Fax form to CASG Central Unit within 2 business days of knowledge of event
Report SAE to site IRB (per local IRB guidelines)
Log SAE in Adverse Event CRF
Submit original report within 5 business days to the CASG CU
If warranted, follow up to the initial SAE report should be completed and submitted to the CASG CU within 7 business days of initial report.

The CASG central unit will:

Submit the SAE report to DMID Project Officer, PPD, Gilead Sciences, Inc. and the CASG Biostatistics Unit within 2 business days of receipt of fax.

If other Adverse event

The site will:

Document event on the appropriate AE CRF
Report AE to site IRB (per local IRB guidelines)

Submit AE reports monthly to the CASG Central Unit or as requested

The CASG central unit will:

Report to the DMID Project Officer, and to CASG Biostatistics Unit

8.3.4. Pregnancy

Cidofovir is a pregnancy category C drug and has been known to cause hypospermia and embryotoxicity and maternal toxicity in rats and rabbits, any pregnancy that occurs in women who are treated under this protocol must be reported to the CASG Central Unit within 48 hours of the site's knowledge of the pregnancy. The pregnancy should be reported on the SAE reporting form. The CASG Central Unit will notify DMID within 24 hours of receiving the report. The site will be given any specific instructions on follow-up of the pregnancy at that time. All pregnancies will be followed for outcome.

8.3.5. Expected Adverse Events

Side effects that have been noted in previous recipients of cidofovir range from very mild to critical. Milder side effects are diarrhea, headache, loss of appetite, nausea, vomiting, generalized weakness, fever, chills, sore throat and loss of strength. More serious side effects are nephrotoxicity, Fanconi’s syndrome and decreases in serum bicarbonate associated with renal tubular damage, proteinuria, elevated serum creatinine, glycosuria, hematuria, urinary incontinence, UTI, and acute renal failure. Neutropenia, granulocytopenia, thrombocytopenia and anemia have also been known to occur. Adverse events that are related to the eye are amblyopia, conjunctivitis, eye disorder, iritis, retinal detachment, uveitis, abnormal vision, and hypotonia.

9.0. STATISTICAL METHODS

9.1. General Considerations

This is a Phase I/II, randomized, double-blind, placebo-controlled, sequential dose-escalation study of cidofovir in renal transplant recipients with biopsy or plasma confirmed BKVN. The primary objective for this study is to evaluate the safety and tolerability of 3 dose levels of cidofovir when administered to kidney transplant recipients with BKVN and identify the MTD within this dosage range. The secondary objectives are to evaluate the antiviral effect of three dose levels of cidofovir, define the PK of cidofovir in renal transplant recipients with underlying renal impairment, and correlate PK with the PD of cidofovir. A total of approximately 36 subjects will be enrolled and randomized into 3 dose cohorts, with 12 subjects within each dose cohort. Subjects in Cohorts I, II and III will be randomized in a 2:1 ratio to receive cidofovir or placebo, respectively. Twelve additional subjects (randomized in a 2:1 ratio to receive cidofovir or placebo, respectively) will be enrolled at the MTD and will comprise the group for PK and efficacy analysis. Dose escalation and accrual to the next cohort will occur only after fully evaluating subjects for DLTs, and will continue until DLTs stop further escalation.

Table 3: Dose Cohorts and the number of subjects in each

Dose Cohorts	N	Placebo Controls	Totals	Cumulative Total at each stage
0.25 mg/kg	8	4	12	12
0.50 mg/kg	8	4	12	24
1.0 mg/kg	8	4	12	36
Additional Subjects	8	4	12	48
Total	32	16	48	

9.2. Sample Size Justification

9.2.1. Safety Evaluation

The primary objective of this study is to determine safety and tolerability of cidofovir in renal transplant recipients based on adverse events. All patients who receive at least one dose of study medication will be included in the safety analysis (section 9.4.1). Specific safety events to be monitored will include the following: death, or serious adverse events related to study drug, including deterioration in renal function from baseline, decline in absolute neutrophil count to less than 500 per cubic mm, and ocular complications (hypotony, uveitis). A decrease in glomerular filtration rate of > 50% from baseline or ANC < 500 per μL will be considered as dose limiting toxicities for the purposes of escalation to the next dose cohort (DLT). For purposes of evaluating the safety of cidofovir, we will assume that an acceptable DLT rate is less than or equal to 25%. Table 4 describes the statistical properties of the escalation scheme.

Table 4: Evaluation of the design with respect to probability of escalating

True DLT Rate	Probability of Escalation at any cohort	Probability of 2 DLT's at any cohort	Probability of 3 or more DLT's at any cohort
0.05	0.94	0.05	0.01
0.10	0.81	0.15	0.04
0.15	0.65	0.24	0.11
0.20	0.50	0.29	0.20
0.25	0.37	0.31	0.32
0.30	0.26	0.30	0.45
0.35	0.17	0.26	0.57

Since the relatedness of many SAEs, including unexpected deaths, due to the study medication will be difficult to assess, the criteria for stopping any dose cohorts will not be based solely on defined DLTs. The DSMB will evaluate all medical and statistical factors in assessing whether

the safety profile is of sufficient concern to warrant halting dose escalation and/or trial termination.

Although the sample size estimate at each dose cohort is based on the escalation decision as described above, the sample size also allows for reasonable estimation of adverse event rates and efficacy at the recommended dose MTD . For example, upon completion of this trial, if the 1.0 mg/kg dose becomes the MTD then there will be a approximately 48 study participants randomized to either cidofovir (n=32) or placebo (n=16). For each cohort, the sample size of 8 will allow 15.3% estimated standard error for adverse event rate for treatment group, and sample size of 4 in control group will allow 21.7% estimated standard error for adverse event rate if we assume that the adverse event rate is less than or equal to 25%. When all four cohorts are combined, a sample size of 32 will give an overall 7.7% estimated standard error for adverse event rate for the dosing group. A sample size of 16 will give a 10.8 % estimated standard error for adverse event rate for the control group.

9.2.2. Efficacy Evaluation

Based on the small sample size and study design, the present study has limited power to detect efficacy of cidofovir for treatment of BKVN. The primary goal is to define safety, tolerability, and maximum tolerated dose of cidofovir in this population of kidney transplant recipients who are at high risk for nephrotoxicity. This study will allow us to obtain preliminary estimates of efficacy of cidofovir based on virologic endpoints. Efficacy will be defined as a 1-log decrease in viral load at 5 weeks from baseline based on biological response in patients who have received 5 weeks of treatment.

9.3. Treatment Assignment and Blinding

This is a randomized, placebo-controlled dose escalation study with the identity of the treatment in the double-blind arms of this study unknown to the investigator, subject, CASG Central Unit, NIAID/DMID, and employees of Gilead Sciences, Inc. conducting this study. The biostatistician will be responsible for generating the randomization schema for this study. Study participants will be randomized in a 2:1 ratio to receive cidofovir or placebo in a blinded fashion in Cohorts I, II and III. After each dose cohort has been fully enrolled, the DSMB will review safety and

toxicity data. A similar randomization procedure described for cohort I will be utilized for cohorts II and III. However, Patient (s) who withdraw or die (not-treatment related) would be replaced in the same regimen in order to keep the study design balanced.

9.4. Safety and Efficacy Analyses

9.4.1. Safety Analysis

All study participants randomized into the study who have received any amount of study medication or placebo will be included in the safety analysis. Adverse events will be summarized by calculating numbers and proportions of study participants exhibiting adverse events for each body system. The proportions of study participants who have experienced Grade 2 + toxicity will be presented by treatment group for each cohort. Estimates of adverse event rates will be calculated for each of the cohorts along with exact 95% confidence intervals. In this analysis, repeated reports by the same patient, of the same adverse event are counted only once; however, tables will also be prepared that show numbers of events, rather than persons. Descriptive analysis will apply to safety lab tests for each cohort including presentation of baseline measurement, and the changes from baseline after treatment.

9.4.2. Efficacy Analysis

In addition to safety, all study participants will be followed with regard to efficacy. If the subjects discontinue or die before completion of all 5 weeks of treatment (4 doses) then they will be listed in a separate efficacy analysis. Viral loads will be measured at multiple time points. Mean and median log changes of viral load from baseline will be presented for the treatment group and the control group separately for each cohort. Descriptive analysis will also be applied to 1) the percentage of study participants who achieve a negative BK virus urine/plasma PCR between baseline and end of treatment; 2) the percentage of study participants who have reduction in urine/plasma BK virus load by quantitative PCR between baseline and end of treatment and 3) time to reduction in BK virus urine/plasma by PCR. The time until reduction (or a 1-log reduction) will be recorded for each patient. Kaplan-Meier curves will be computed and plotted for overall and for each cohort by treatment group. Additionally, statistical analysis for allograft rejection will be descriptive. The frequencies and the percentage of allograft rejection

(percentage of allograft rejection is the proportion of subjects with allograft rejection from the total number of subjects in the treatment group) will be presented for each dose cohort and for each treatment group, cidofovir and placebo, separately.

Statistical analysis will be done using StatXact 4.0 for exact statistical methods and SAS 9.0.

9.4.3. Subject Disposition

A detailed description of patient disposition will include:

- A summary of data on patient discontinuation
- A summary of data on overall qualification status of all study participants
- An account of all identified protocol deviations

All study participants entered in the study will be accounted for in the summation. The number of study participants who do not qualify for analysis, who die, or who discontinue before treatment begins will be specified.

9.5. Pharmacokinetic and Pharmacodynamic Analyses

The purpose of the data collection schemes outlined above is to enable pharmacokinetic modeling of the concentration-time results with incorporation of urine data. The pharmacokinetic parameters will then be used to explore pharmacodynamic relationships.

Cidofovir plasma and urine concentrations will be measured at the University of Alabama at Birmingham CASG Core Pharmacology Laboratory. The assays will be developed using high performance liquid chromatography with mass spectrometry detection (LC/MS). The lower limit of detection will be in the range of 1-5 ng/mL with <10% variability at the low and high end of the standard curves. First-dose and multiple-dose pharmacokinetic data will be modeled using Bayesian estimation. The Bayesian prior will be developed using either literature data or, preferably, parameters and variability derived from the intensive pharmacokinetic evaluation will be used. The intensive concentration-time data may be analyzed using a noncompartmental

approach or modeled using generalized least squares (GLS) or maximum likelihood parameter estimation. The model will likely be a two-compartment, linear elimination model parameterized for total clearance (CL_T). Within this model, it is possible to use CLcr from each subject at the time of the pharmacokinetic data collection to adjust CL_T thereby allowing us to simultaneously fit the plasma and urine concentration-time data. These models also allow for fitting of non-uniform data, so the first- and multiple-dose concentration-time data can also be co-modeled.

Pharmacodynamic analyses will consist of comparing various pharmacokinetic parameters (AUC, C_{max}, etc.) with changes in BK virus load in plasma and urine. These relationships will be explored using maximum effect models (E_{max}) and linear models. The goal of the pharmacodynamic analyses is to try to determine the dose (and total drug exposure) required to produce the maximum change in plasma and urine BK virus load.

10.0. STUDY AND INVESTIGATOR REQUIREMENTS

10.1. Protocol Adherence

Each investigator must adhere to the protocol as detailed in this document and agree that any changes to the protocol must be approved by the CASG and NIAID/DMID prior to seeking approval from the IRB/IEC. Each investigator will be responsible for enrolling only those study participants who have met protocol eligibility criteria.

A protocol deviation is any noncompliance with the clinical trial protocol, Good Clinical Practice (GCP), or protocol-specific Manual of Procedures requirements. The noncompliance may be either on the part of the subject, the investigator, or the study site staff. As a result of deviations, corrective actions are to be developed by the site and implemented promptly.

These practices are consistent with Good Clinical Practice (ICH E6):

Section 4.5 Compliance with Protocol, sections 4.5.1, 4.5.2, and 4.5.3
Section 5.1 Quality Assurance and Quality Control, section 5.1.1
Section 5.20 Noncompliance, sections 5.20.1, and 5.20.2.

It is the responsibility of the site PI/study staff to use continuous vigilance to identify and report deviations within 5 working days of identification of the protocol deviation, or within 5 working days of the scheduled protocol-required activity. All deviations must be promptly reported to CASG Central Unit for submission to NIAID/DMID

All protocol deviations, as defined above, must be addressed in study subject source documents. A completed copy of the DMID Protocol Deviation (PD) Form (PPD or IDES form) must be maintained in the Regulatory File, as well as in the subject's source document. Protocol deviations must be sent to the local IRB/IEC per their guidelines. The site PI/study staff is responsible for knowing and adhering to their IRB requirements.

10.2. Quality Assurance and Quality Control

The CASG Central Unit will assure protocol and regulatory compliance and data quality by frequent contact and open dialogue with site personnel, thorough review of submitted SAE and CRFs and scheduling monitor visits and reviewing monitoring reports. Study participants will receive all drug administrations by a qualified designee under the supervision of the principal investigator listed on the Statement of Investigator (FDA Form 1572). Collection of accurate, consistent, and reliable data is the responsibility of the principal investigator at each site and will be ensured through the use of standard practices and procedures. Each site is asked to implement a quality control program.

10.3. Case Report Forms

CRFs will be supplied by the CASG. All submitted CRFs will be reviewed for completeness by the CASG Adult Clinical Administrator and/or a designee. Study monitors will compare the case report forms to the source document, and queries will be generated for site resolution with changes to be incorporated into the database by the Biostatistics unit. Data management personnel will utilize inter- and intra-CRF validation and logic edit checks that will be run periodically and will be automatically included into the computerized data query system, which tracks the details of the query and its response. In addition, a supplemental quality control monitoring report will be provided to site study personnel on a quarterly basis: The Data Quality and Monitoring Report, which tracks accrual, CRF delinquency rates and endpoint completeness. Specific instructions for CRF completion and query resolution will be detailed in the MOP.

10.4. Source Documentation

Documentation of source data is necessary for the reconstruction, evaluation and validation of clinical findings, observations, and other activities during a clinical trial. Source documentation serves to substantiate the integrity of trial data, confirms observations that are recorded, and confirm the existence of study participants. This standard also serves to ensure data quality by creating audit trails and enabling verification that data re present, complete, and accurate. CASG and DMID studies will be monitored using these standards.

Sites that are participating in this trial should consult the MOP and DMID/NIAID Source Document Standards Version 2.0 (most current version) for specific instruction and forms.

Local, state, institution, institutional review board (IRB)/independent ethics committee (IEC) policies and procedures may be different from those stated in this standard. The site should refer to local, state, institution, IRB/IEC policies and procedures and follow them if they are more stringent than the DMID Standards.

According to the ICH GCP 4.9: "The investigator should ensure the accuracy, completeness, legibility, and timeliness of the data reported to the sponsor in the CRFs and in all required reports. Data reported on the CRFs, which are derived from source documents, should be consistent with the source documents or discrepancies should be explained."

10.5. Site Monitoring

Site Monitoring will be conducted to ensure that human subject protection, study procedures, laboratory procedures, study intervention administration, and data collection processes are of high quality and meet sponsor, CGP/ICH, and regulatory guidelines, and that the study is conducted in accordance with the protocol and study manuals. NIAID/ DMID, the sponsor of this study, or its designee will conduct site monitoring visits as detailed in the monitoring plan or in the Manual of Procedures.

Site visits will be made at standard intervals as defined by CASG and NIAID/DMID and may be made more frequently as directed by CASG or NIAID/DMID. Monitoring visits will include, but are not limited to, review of regulatory files, accountability records, case report forms, informed consent forms, medical and laboratory reports, and protocol compliance. Study monitors will meet with investigators to discuss any problems, actions to be taken, and to document visit findings and discussions.

The investigator will permit authorized representatives of CASG, NIAID, FDA, IRB and industry collaborator Gilead Sciences Inc. to inspect facilities and records relevant to this study.

10.6. Data Handling and Record Retention

Records and documents pertaining to the conduct of this study, including CRFs, source documents, consent forms, laboratory test results, and medication inventory records, must be retained by the investigator for at least 15 years. No study records shall be destroyed without prior authorization from the CASG and NIAID/DMID.

10.7. Informed Consent

A sample informed consent document for this study is provided in the MOP and also separately. This sample consent may be modified to adhere to local IRB/Ethics Committee language. Prior to submitting the site specific informed consent to the local IRB/Ethics Committee, the CASG Central Unit must receive, review and approve the consent form, assuring that no required language has been altered.

The process of obtaining informed consent must be documented in the medical records, clinic chart and research chart. The consent form must be signed and dated by the study participant or legal representative before participation in the study. A copy of the signed consent form must be provided to the study participant. Signed consent forms must remain in each study participant's study file and must be available for verification by study monitors at any time. Counseling related to pregnancy prevention and testing will also be documented. All informed consents must meet site specific institutional HIPAA compliance.

10.8. IRB/IEC Approval

This protocol, the patient information sheet, informed consent document, relevant supporting information, and all types of patient recruitment or advertisement information must be submitted to the IRB/IEC for review and must be approved before the study is initiated. Protocol amendments will be generated by the sponsor. Any amendments to the protocol must also be approved by the IRB/IEC prior to implementing changes in the study. No study medications will be shipped to a site until all required regulatory documents have been received by the CASG Central Unit.

The investigator is responsible for keeping the IRB/IEC appraised of the progress of the study and of any changes made to the protocol as deemed appropriate, but in any case at least once a year. The investigator must also keep the IRB/IEC informed of any significant adverse events.

10.9. Anonymity and Confidentiality

The information obtained during the conduct of this clinical study is confidential, and disclosure to third parties other than those noted below is prohibited. The results of the research study may be published, but study participant's names or identities will not be revealed. Records will remain confidential. To maintain confidentiality, the principal investigators at each site will keep records in locked cabinets and the results of tests will be coded to prevent association with volunteers' names. Data entered into computerized files will be accessible only by authorized personnel directly involved with the study and will be encoded. The NIAID/DMID, CASG, and Gilead Sciences Inc. may use information obtained during the conduct of this study in connection with the development of the study drug. The study investigator is obliged to provide the NIAID/DMID, CASG, and Gilead Sciences Inc. with complete test results and all data developed in this study. The NIAID/DMID, CASG or Gilead Sciences Inc. may disclose this information to appropriate regulatory authorities as deemed necessary by the NIAID/DMID, CASG or Gilead Sciences Inc. Patient-specific information may be provided to other appropriate medical personnel only with the study participant's permission. To ensure compliance with current ICH guidelines, data generated by this study must be available for inspection upon request by representatives of national and local health authorities, the NIAID/DMID, CASG, Gilead Sciences Inc. and the IRB/IEC for each study site.

10.10. Future Use of Specimens

Some of the specimens obtained from study participants during this study (urine and serum) will be stored indefinitely in the CASG Central Laboratory at the University of Alabama at Birmingham and may be used in future BK virus research. These specimens will be labeled with a code number and not with the study participant's name. At the time of consent for study participation, study participants will have the opportunity to either agree or decline to have their specimens used in future BK virus research. The study participant will indicate his/her preference

by initialing the appropriate line or checking the appropriate box of the Consent Form in the section entitled, "Future Use of Specimens". Non-protocol designated, future testing of samples will be performed only on samples from study participants who have consented for future testing of samples. These specimens will only be utilized to better understand the natural history of this disease or improve diagnosis.

A repository for residual samples will be established according to OHRP guidelines ensuring that codes or other personally identifying links will not be distributed to future researchers.

The specimens will be stored indefinitely in the CASG Central Laboratory at the University of Alabama at Birmingham. BK virus specimens from study participants will be labeled and coded without study participant's identifiers. If the study participant has indicated in the signed consent form that he/she does not agree to allow the future use of specimens for future BK virus research, then his/her specimens will be destroyed at the completion of the study.

10.11. Health Insurance Portability and Accountability Act of 1996 (HIPAA)

Each institution involved in this study will adhere to the Health Insurance Portability and Accountability Act of 1996 (HIPAA) as applicable according to individual institutional policies.

11.0. REFERENCES

1. Shah KV, Daniel RW, Warszawski RM. High prevalence of antibodies to BK virus, an SV40-related papovavirus, in residents of Maryland. J Infect Dis 1973; 128:784-7.
2. Chester PM, Heritage J, D.J. M. Persistence of DNA sequences of BK virus and JC virus in normal human tissues and in diseased tissues. J Infect Dis 1983; 147(4):676-84.
3. Ling PD, Lednicky JA, Keitel WA, et al. The dynamics of herpesvirus and polyomavirus reactivation and shedding in healthy adults: a 14-month longitudinal study. J Infect Dis 2003; 187:1571-80.
4. Gardner SD, MacKenzie EF, Smith C, Porter AA. Prospective study of the human polyomaviruses BK and JC and cytomegalovirus in renal transplant recipients. J Clin Pathol 1984; 37:578-86.
5. Hogan TF, Borden EC, McBain JA, Padgett BL, Walker DL. Human polyomavirus infections with JC virus and BK virus in renal transplant patients. Ann Intern Med 1980; 92:373-8.
6. Hirsch HH, Knowles W, Dickenmann M, et al. Prospective study of polyomavirus type BK replication and nephropathy in renal-transplant recipients. N Engl J Med 2002; 347:488-96.
7. Binet I, Nickeleit V, Hirsch HH, et al. Polyomavirus disease under new immunosuppressive drugs: a cause of renal graft dysfunction and graft loss. Transplantation 1999; 67:918-22.
8. Boubenider S, Hiesse C, Marchand S, Hafi A, Kria F, Charpentier B. Post-transplantation polyomavirus infections. J Nephrol 1999; 12:24-9.
9. Howell DN, Smith SR, Butterly DW, et al. Diagnosis and management of BK polyomavirus interstitial nephritis in renal transplant recipients. Transplantation 1999; 68:1279-88.
10. Pappo O, Demetris AJ, Raikow RB, Randhawa PS. Human polyoma virus infection of renal allografts: histopathologic diagnosis, clinical significance, and literature review. Mod Pathol 1996; 9:105-9.
11. Randhawa PS, Finkelstein S, Scantlebury V, et al. Human polyoma virus-associated interstitial nephritis in the allograft kidney. Transplantation 1999; 67:103-9.
12. Andrews CA, Shah KV, Daniel RW, Hirsch MS, Rubin RH. A serological investigations of BK virus and JC virus infections in recipients of renal allografts. J Infect Dis 1988; 158:176-81.
13. Nickeleit V, Hirsch HH, Binet IF, et al. Polyomavirus infection of renal allograft recipients: from latent infection to manifest disease. J Am Soc Nephrol 1999; 10:1080-9.
14. Hirsch HH. Polyomavirus BK nephropathy: a (re-)emerging complication in renal transplantation. Am J Transplant 2002; 2:25-30.
15. Nickeleit V, Klimkait T, Binet IF, et al. Testing for polyomavirus type BK DNA in plasma to identify renal-allograft recipients with viral nephropathy. N Engl J Med 2000; 342:1309-15.

16. Limaye AP, Jerome KR, Kuhr CS, et al. Quantitation of BK virus load in serum for the diagnosis of BK virus-associated nephropathy in renal transplant recipients. J Infect Dis 2001; 183:1669-72.
17. Vats A, Shapiro R, Singh Randhawa P, et al. Quantitative viral load monitoring and cidofovir therapy for the management of BK virus-associated nephropathy in children and adults. Transplantation 2003; 75:105-12.
18. Kadambi PV, Josephson MA, Williams J, et al. Treatment of refractory BK virus-associated nephropathy with cidofovir. Am J Transplant 2003; 3:186-91.
19. Hirsch HH, Steiger J. Polymovirus BK. Lancet Infect Dis 2003; 3(10):611-23.
20. Ramos E, Drachenberg CB, Papadimitriou JC, et al. Clinical course of polyoma virus nephropathy in 67 renal transplant patients. J Americ Soc Nephrol 2002; 13:2145-51.
21. Drachenberg CB, Beskow CO, Cangro CB, et al. Human polyoma virus in renal allograft biopsies: morphological findings and correlation with urine cytology. Hum Pathol 1999; 30:970-7.
22. Andrei G, Snoeck R, Vandeputte M, De Clercq E. Activities of various compounds against murine and primate polyomavirusesAntimicrobial Agents & Chemotherapy. Antimicrob Agents Chemother 1997:587-93.
22.b. Farasati NA, Shapiro R, Vats A, Randhawa P. Effect of leflunomide and cidofovir on replication of BK virus in an in vitro culture system. Transplantation. 2005 Jan 15;79(1):116-8.
23. Bjorang O, Tveitan H, Midtvedt K, Broch LU, Scott H, Andersen PA. Treatment of polyomavirus infection with cidofovir in a renal-transplant recipient. Nephrol Dial Transplant 2002; 117:2023-5.
24. Scantlebury V, Shapiro R, Randhawa P, Weck K, Vats A. A method of treatment for BK virus-associated transplant nephropathy. Graft 2002; 5:S82-S87.
25. Keller LS, Peh CA, Nolan J, Bannister KM, Clarkson AR, Faull RJ. BK transplant nephropathy successfully treated with cidofovir. Nephrol Dial Transplant 2003; 18:1013-4.
26. Vats A. Abstract ATC 2003.
27. Herman. Abstract ATC 2003.
28. De Clercq E. Acyclic nucleoside phosphonates in the chemotherapy of DNA virus and retrovirus infections. Intervirology 1997; 40:295-303.
29. De Luca A, Giancola ML, Ammassari A, et al. Cidofovir added to HAART improves virological and clinical outcome in AIDS-associated progressive multifocal leukoencephalopathy. AIDS 2000; 14:F117-21.
30. Cundy KC, Petty BG, Flaherty J, et al. Clinical pharmacokientics of cidofovir in human immunodeficiency virus-infected patients. Antimicrob Agents Chemother 1995; 39:1247-52.
31. Cundy KC, Barditch-Crovo P, Petty BG, et al. Clinical pharmacokinetics of 1-[((S)-2-hydroxy-2-oxo-1,4,2-dioxaphosphorinan-5-yl)methyl]cytosine in human immunodeficiency virus-infected patients. Antimicrob Agents Chemother 1999; 43:271-7.
32. Cundy KC, Bidgood AM, Lynch G, Shaw JP, Griffin L, Lee WA. Pharmacokinetics, bioavailability, metabolism, and tissue distribution of cidofovir (HPMPC) and cyclic HPMPC in rats. Drug Metab Dispos 1996; 24:745-52.

33. Cundy KC, Li ZH, Hitchcock MJ, Lee WA. Pharmacokinetics of cidofovir in monkeys. Evidence for a prolonged elimination phase representing phosphorylated drug. Drug Metab Dispos 1996; 24:738-44.
34. Scott R, Pavesio C. Ocular side-effects from systemic HPMPC (Cidofovir) for a non-ocular cytomegalovirus infection. Am J Ophthal 2000; 130(1):126-7.
35. Ljungman P, Ribaud P, Eyrich M, et al. Cidofovir for adenovirus infections after allogeneic hematopoietic stem cell transplantation: a survey by the Infectious Diseases Working Party of the European Group for Blood and Marrow Transplantation. Bone Marrow Transplant 2003; 31:481-6.
36. Ljungman P, Deliliers GL, Platzbecker U, et al. Cidofovir for cytomegalovirus infection and disease in allogeneic stem cell transplant recipients. The Infectious Diseases Working Party of the European Group for Blood and Marrow Transplantation. Blood 2001; 97:388-92.
37. Chakrabarti S, Collingham KE, Osman H, Fegan CD, Milligan DW. Cidofovir as primary pre-emptive therapy for post-transplant cytomegalovirus infections. Bone Marrow Transplant 2001; 28:879-81.
38. Chou S, Lurain NS, Thompson KD, Miner RC, Drew WL. Viral DNA polymerase mutations associated with drug resistance in human cytomegalovirus. Journal of Infectious Diseases. 2003; 188:32-9.
39. Erice A. Resistance of human cytomegalovirus to antiviral drugs. Clinical Microbiology Reviews. 1999; 12:286-97.
40. Buehrig CK, Lager DJ, Stegall MD, et al. Influence of surveillance renal allograft biopsy on diagnosis and prognosis of polyomavirus-associated nephropathy. Kidney International. 2003; 64:665-73.
41. Brody SR, Humphreys MH, Gambertoglio JG, Schoenfeld P, Cundy KC, Aweeka FT. Pharmacokinetics of cidofovir in renal insuffieciency and in continuous ambulatory peritoneal dialysis or high-flux hemodialysis. Clin Pharmacol Ther 1999; 65:21-8.

APPENDIX A. Schedule of Procedures

Study Procedures	Screening Period Day (-) 21 - 0	Day 0		Day 7 ± 1		Day 14 ± 2	Day 21 ± 1		Day 28 ± 2	Day 35 ± 1		Day 42 ± 2	Day 49 ± 3	Day 120 ± 14
		Baseline Pre – dosing[11]		Pre – dosing[11]			Pre – dosing[11]			Pre – dosing[11]				
Obtain informed consent	X													
Randomize Patient		X												
Medical, Surgical and Transplant History	X													
Physical Exam & Vital signs [1]	X	X		X			X			X			X	
Pregnancy Test [2]	X	X		X			X			X			X	
Urine Dipstick[3] (Urine Creatinine and urine protein [10])	X	X		X			X			X			X	
Safety Labs[4,12]	X[4]	X[4]		X[4]		X[12]	X[4]		X[12]	X[4]		X[12]	X[4]	
Plasma BK viral load by PCR	X	X		X			X			X			X	
Urine BK viral load by PCR	X	X		X			X			X			X	
Urine for Future CMV Testing[12]		X											X	
Serum Trough Levels – immunosuppressants[5]	←												→	
Cidofovir/Placebo Infusion			X		X			X			X			
Renal biopsy[6]		←											→	
Concomitant Medications	←												→	
Adverse Events		←											→	
Concomitant Immunosuppressive Therapy	←												→	
Plasma for PK[7] a – Limited Data Sample Collection b – Intensive Data Sample Collection		X[7a or b]								X[7a or b]				
24 hour Urine for PK[8] (MTD only)		X								X				
Ocular Exam[9]	X[9]	←							→	X[9]		←	→	
Pregnancy Follow-up Phone Call														X
Pregnancy Counseling	X			X			X			X			X	

[1] A comprehensive physical with vital signs: weight, blood pressure (BP), heart rate (HR), respiratory rate (RR), and temperature (T) will be done during the screening period,(-)21 to 0 days) before randomization; subsequent exams will be brief physicals with vital signs and weight.

[2] Pregnancy test: to be done within 48 hours of starting study drug for all women who are not post menopausal if positive, these women will be excluded from this study. Also pregnancy tests will be conducted within 48 hours before each subsequent dose, if positive patient is excluded from receiving additional study drug. A final pregnancy test will be done at Day 40±3.

[3] Urine dipstick test (pH, glucose, protein, nitrite, leukocyte esterase, occult blood, ketones, bilirubin, urobilinogen) and spot urine test for quantitative urine protein/creatinine ratio.

[4] Safety Labs :
Complete blood cell count with differential [(manual or automated) platelet count, hemoglobin, hematocrit ,RBC, WBC] electrolytes (sodium, potassium, chloride, bicarbonate), blood urea nitrogen (BUN), serum creatinine, calcium, magnesium, phosphorous, total protein, glucose, albumin, total bilirubin, ALT, AST, Alkaline phosphatase,. Calculate GFR and calculate ANC at screening (day -21 – 0), prior to dosing at baseline (day 0), days 7±1, 21±1, 35±1 and after dosing on day 49±3.

[5] Serum Trough Levels of Immunosuppressants; to be collected when and if tests conducted for standard of care.

[6] Renal Biopsy: performed in any subject who develops a > 50% reduction from baseline in glomerular filtration rate while on study. If a renal biopsy is conducted at any time (pre-study as part of standard of care or during study) any remaining specimens and slides that are obtainable will be sent to the CASG Central Laboratory.

[7] Plasma for PK:

[7a] Limited Sample Data Collection (for dose cohorts **below** MTD)
PKs for the first dose pharmacokinetics
A pre-dose blood sample will be collected within 20 minutes of beginning the infusion. After the dose is administered, a post-infusion blood sample should be drawn (within 15 minutes after infusion). Blood my be drawn from the same arm in which the study drug was administered as long as the blood draw is performed at 15 minutes after the study drug is infused. Additional blood samples should be obtained at 2 hours ± 15 min. and 4 hours ± 15 min after the end of the infusion.

[7b] Intensive Sample Data Collection (for dose cohort **at** MTD)
A pre-dose blood sample will be collected within 20 minutes of beginning the infusion. After the dose has infused, a post-infusion blood sample should be drawn (within 15 minutes after infusion). PKs will be drawn from the opposite arm of the infusion. Additional blood samples for determination of cidofovir in plasma should be collected at time 1hour ± 15 min, 2 hours ± 15 min, 3 hours ± 15 min, and 4 hours ± 15 min post infusion.

[8] Urine for PK: A 24 hour urine collection will be performed at the each pharmacokinetic evaluation period. Participants are to void prior to receiving the study drug. They will be given 2 collection containers; one for the first 12 hours following the dose and one for the second 12 hours. Participants will be instructed to void into the respective containers for 24 hours following the first dose of cidofovir. This schedule of urine collection will be performed only in patients at the MTD/1.0 mg/kg dose at: 1st dose of study drug (day 0), and at last dose of study drug (day 35).

[9] Ocular exam: Ophthalamologic monitoring exams will be done by an eye care specialist. Ocular pressure will be measured and a slit-lamp examination will be done on the following schedule: within 3 weeks prior to initial study drug infusion and within 2 week after the last dose. Ophthalmologic exam for any ocular symptoms may occur at any time during the study if clinically indicated.

[10] Urine protein and urine creatinine - to be obtained within 48 hours of each dose of cidofovir

[11] Tests at pre-dosing are to be done within 48 hours of starting drug unless otherwise specified.

[12] Safety tests conducted 1 week post each study drug administration will include: Electrolytes – sodium, potassium, chloride, bicarbonate; blood urea nitrogen, serum creatinine, and spot urine for quantitative creatinine and protein..
