# Supplementary material for: A randomized, placebo‐controlled, dose‐escalation phase I/II multicenter trial of low‐dose cidofovir for BK polyomavirus nephropathy
Source: Transpl Infect Dis. 2024 Sep 3;26(6):e14367. doi: 10.1111/tid.14367 (PMC11666883; doi:10.1111/tid.14367)
Supplement: Supplementary file 3 — Visual Abstract Supporting information [file TID-26-e14367-s001.pptx]

## Slide 1
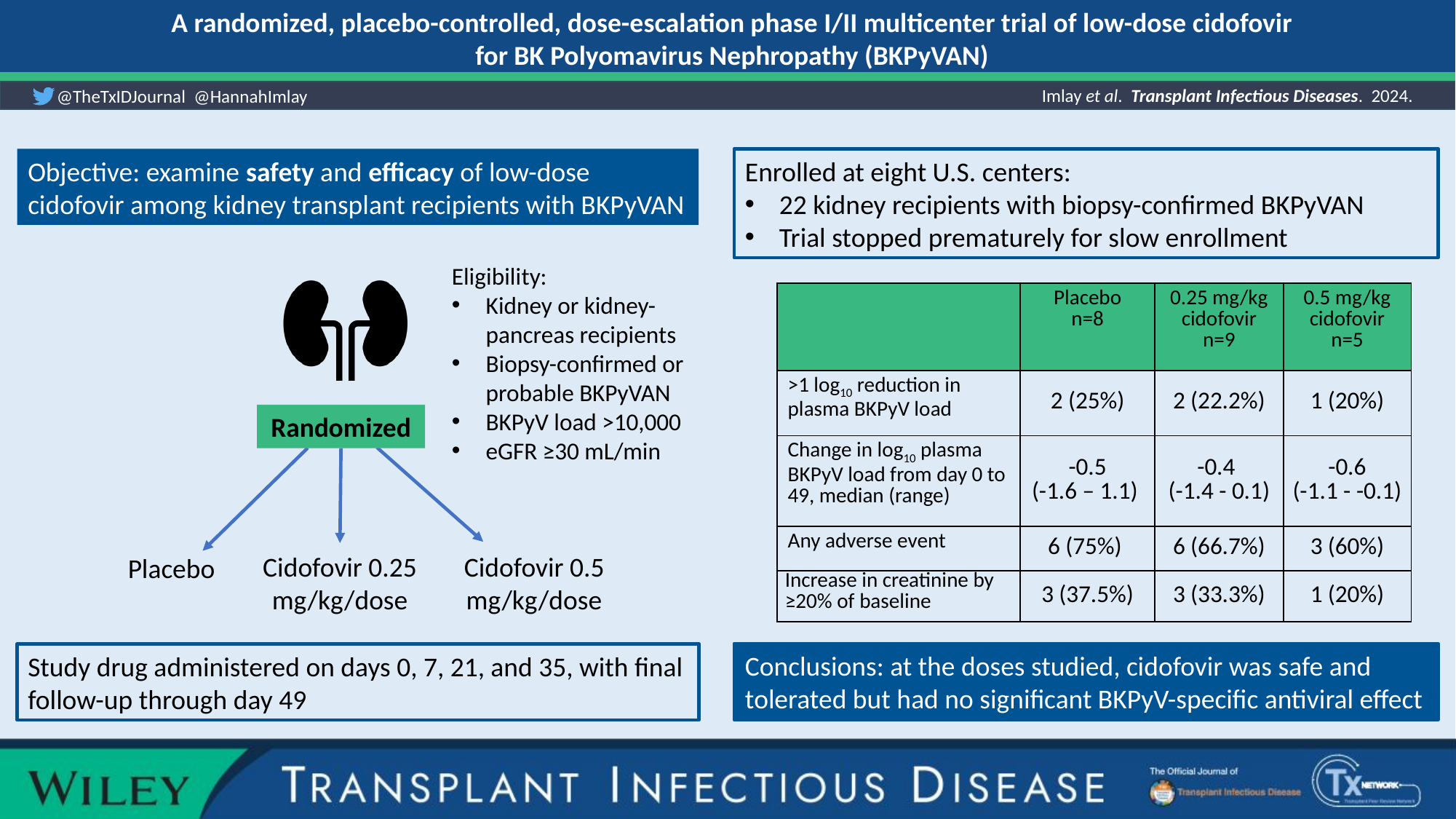

A randomized, placebo-controlled, dose-escalation phase I/II multicenter trial of low-dose cidofovir for BK Polyomavirus Nephropathy (BKPyVAN)
Imlay et al. Transplant Infectious Diseases. 2024.
 @TheTxIDJournal @HannahImlay
Objective: examine safety and efficacy of low-dose cidofovir among kidney transplant recipients with BKPyVAN
Enrolled at eight U.S. centers:
22 kidney recipients with biopsy-confirmed BKPyVAN
Trial stopped prematurely for slow enrollment
Eligibility:
Kidney or kidney-pancreas recipients
Biopsy-confirmed or probable BKPyVAN
BKPyV load >10,000
eGFR ≥30 mL/min
| | Placebo n=8 | 0.25 mg/kg cidofovir n=9 | 0.5 mg/kg cidofovir n=5 |
| --- | --- | --- | --- |
| >1 log10 reduction in plasma BKPyV load | 2 (25%) | 2 (22.2%) | 1 (20%) |
| Change in log10 plasma BKPyV load from day 0 to 49, median (range) | -0.5 (-1.6 – 1.1) | -0.4 (-1.4 - 0.1) | -0.6 (-1.1 - -0.1) |
| Any adverse event | 6 (75%) | 6 (66.7%) | 3 (60%) |
| Increase in creatinine by ≥20% of baseline | 3 (37.5%) | 3 (33.3%) | 1 (20%) |
Randomized
Cidofovir 0.25 mg/kg/dose
Cidofovir 0.5 mg/kg/dose
Placebo
Conclusions: at the doses studied, cidofovir was safe and tolerated but had no significant BKPyV-specific antiviral effect
Study drug administered on days 0, 7, 21, and 35, with final follow-up through day 49
